# Supplementary material for: Structure-based discovery of hydrocarbon-stapled paxillin peptides that block FAK scaffolding in cancer
Source: Nat Commun. 2025 Feb 28;16:2060. doi: 10.1038/s41467-025-57196-9 (PMC11871066; doi:10.1038/s41467-025-57196-9)
Supplement: Supplementary file 1 — Supplementary Information [file 41467_2025_57196_MOESM1_ESM.pdf]

Supplementary Information -
Reyes et al. Structure-based discovery of hydrocarbon-stapled paxillin peptides that block FAK scaffolding in cancer

Table of Contents:

Supplementary Fig. 1. General synthetic scheme for stapled peptides.
Supplementary Fig. 2. Analytical LC-MS confirms identity of 1907 & 2012 and quantifies >98% analyte purity by 214nm area%.
Supplementary Fig. 3. Surface plasmon resonance (SPR) studies characterize synthetic peptide binding to FAK focal adhesion targeting (FAT) domain.
Supplementary Fig. 4. Fluorescence Polarization (FP) assay characterizes peptide effect on inhibition of the FAK-paxillin interaction.
Supplementary Fig. 5. Hydrocarbon stapling enhances α-helicity of peptides.
Supplementary Fig. 6. HSQC-NMR peak integration studies identify the FAT helix 2-3 binding site as the higher affinity site for peptide 1907 compared to the helix 1-4 site.
Supplementary Fig. 7. Stapled peptide 1907 displays binding at both FAT helix 2-3 and helix 1-4 sites at high concentrations.
Supplementary Fig. 8. FAT-1907 X-ray co-crystallography experiments.
Supplementary Fig. 9. Conformational and interaction changes between native paxillin LD2 peptide and stapled peptide 1907 as observed in the X-ray co-crystal structure.
Supplementary Fig. 10. Electron density maps of FAT-1907 X-ray co-crystal structure.
Supplementary Fig. 11. Additional electron density maps of FAT-1907 X-ray co-crystal structure**.**
Supplementary Fig. 12. Immunofluorescence assay showing 2012 cell permeability and de-localization of FAK from focal adhesions.
Supplementary Fig. 13. Immunofluorescence dose response assay showing 2012 induced de-localization of FAK from focal adhesions in SK-MEL-147 cells.
Supplementary Fig 14. Immunofluorescence assay showing 2012 induced de-localization of FAK from focal adhesions in WM88 melanoma cells.
Supplementary Fig 15. Immunofluorescence dose response assay showing 2012 induced de-localization of FAK from focal adhesions in WM88 melanoma cells.
Supplementary Fig. 16. Interaction between FAK and paxillin proteins is disrupted by 2012 treatment.
Supplementary Fig 17. LDH release assay shows even at high micromolar doses of peptide 2012 and 2020 do not result in damage to plasma membrane.
Supplementary Fig. 18. Peptide 2012 induces apoptosis in dose-dependent and time-dependent manner via Annexin V apoptosis time-course study**.**
Supplementary Fig. 19. Peptide 2012 causes dose-dependent reduction in cancer-cell invasion via transwell invasion assay.
Supplementary Fig. 20. Peptide 2012 shows promising plasma and metabolic stability and no signs of toxicity *in vivo*.
Supplementary Fig. 21. Summary Figure: discovery of hydrocarbon-stapled paxillin mimetics with improved anti-cancer efficacy and selectivity compared to first-generation kinase-domain inhibitors.
Supplementary Table. 1. Mass spectral characterization data for stapled peptides.
Supplementary Table 2. Data collection and refinement statistics (molecular replacement) of FAT-1907 X-ray co-crystal structure (PDB 6PW8).
Supplementary Table 3. FAT-1907 (6PW8) interactions ≤ 4 Å at the protein-peptide interface.
Supplementary Table 4. FAT-LD2 (1OW8) interactions ≤ 4 Å at the protein-peptide interface.
Supplementary Table 5. Eurofins KINOMEscan Profiling.

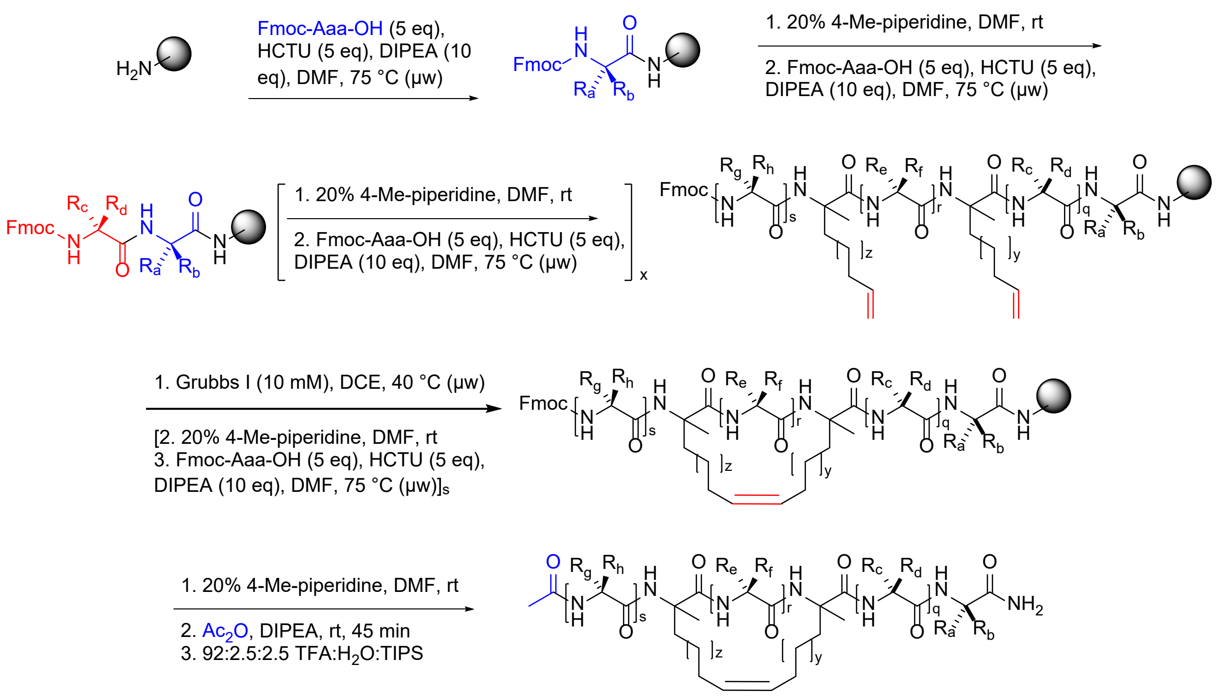

Supplementary Fig. 1. **General synthetic scheme for stapled peptides**. Alpha-helical stapled peptides are assembled C' to N' by serial elongation from a solid support by microwave-assisted solid-phase peptide synthesis. Unnatural olefin-containing amino acids are substituted at strategic positions to allow for favorable ring closing metathesis. Incubation with Grubbs' first-generation catalyst provides solid-supported stapled peptides in near quantitative yields, as a thermodynamic mixture of E and Z isomers (although only the Z isomer is pictured here). Peptides are optionally capped at the amine terminus by conjugation of acetate or myristate. Completed C' amidated peptides are liberated from the solid support by cleavage of the Rink Amide linker with high-TFA cocktail containing scavengers to assist in the concurrent removal of sidechain protecting groups.

1907

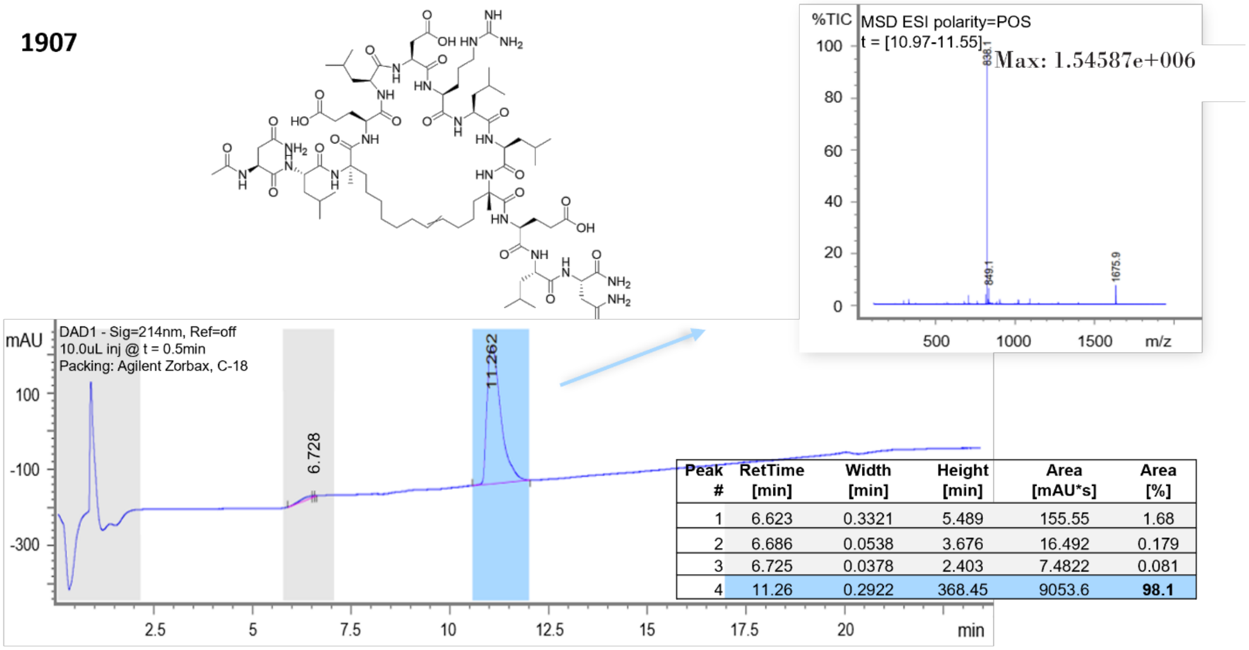

Peak #	RetTime [min]	Width [min]	Height [min]	Area [mAU*s]	Area [%]
1	6.623	0.3321	5.489	155.55	1.68
2	6.686	0.0538	3.676	16.492	0.179
3	6.725	0.0378	2.403	7.4822	0.081
4	11.26	0.2922	368.45	9053.6	**98.1**

2012

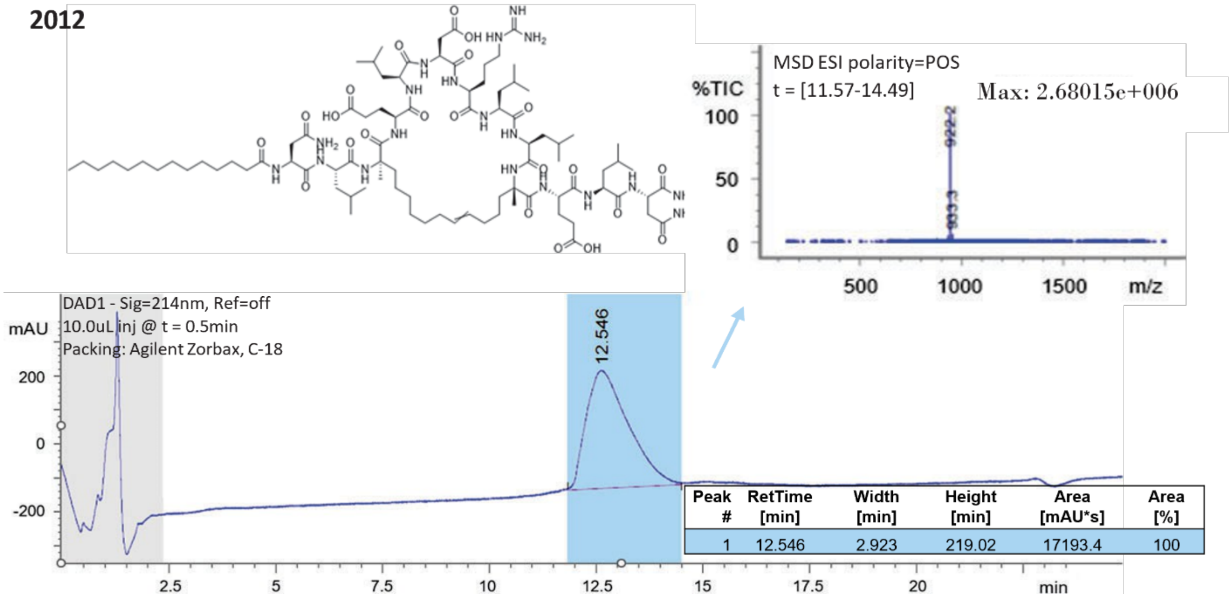

Peak #	RetTime [min]	Width [min]	Height [min]	Area [mAU*s]	Area [%]
1	12.546	2.923	219.02	17193.4	100

HPLC program: Agilent Zorbax C-18, 0.5mL/min, t=0.5min 10uL inject, t=3min gradient elution [10-100% (AcN+0.1%FA in ddH2O+0.1%FA)], t=20min isocratic hold to end of run.

1907:

DAD UV-214nm: peak identified RT=11.262min, automatic integration area% suggests >98% purity.

ESI(positive): scan t=[10.97-11.55], found 838.1, 1675.9, 849.1; C77H131N19O22 M=1673.97: ([M+2H]2+)/2 requires 837.99, [M+2H]2+ requires 1675.97, ([M+Na+H]2+)/2
requires 848.48.

2012:

DAD UV-214nm: peak identified RT=12.546min, automatic integration area% suggests >98% purity.

ESI(positive): scan t=[11.57-14.49], found 922.2, 933.3; C89H155N19O22 M=1842.16: ([M+2H]2+)/2 requires 922.08, ([M+Na+H]2+)/2 requires 932.99.

Supplementary Fig. 2. Analytical LC-MS confirms identity of 1907 and 2012 and quantifies >98% analyte purity by 214nm area%. The peptide structure, chemical formula, molecular weight, and exact mass are listed above the UV-214nm chromatogram. An integration table is included in the bottom right-

hand corner of the chromatogram. The peak of interest is highlighted in blue, and gray highlighted areas represent solvent effects such as injection pressure differential and eluent additive absorption. The %TIC mass spectrum spanning the integrated time period is embedded above the peak of interest and confirms the identities of the analytes as 1907 and 2012 respectively.

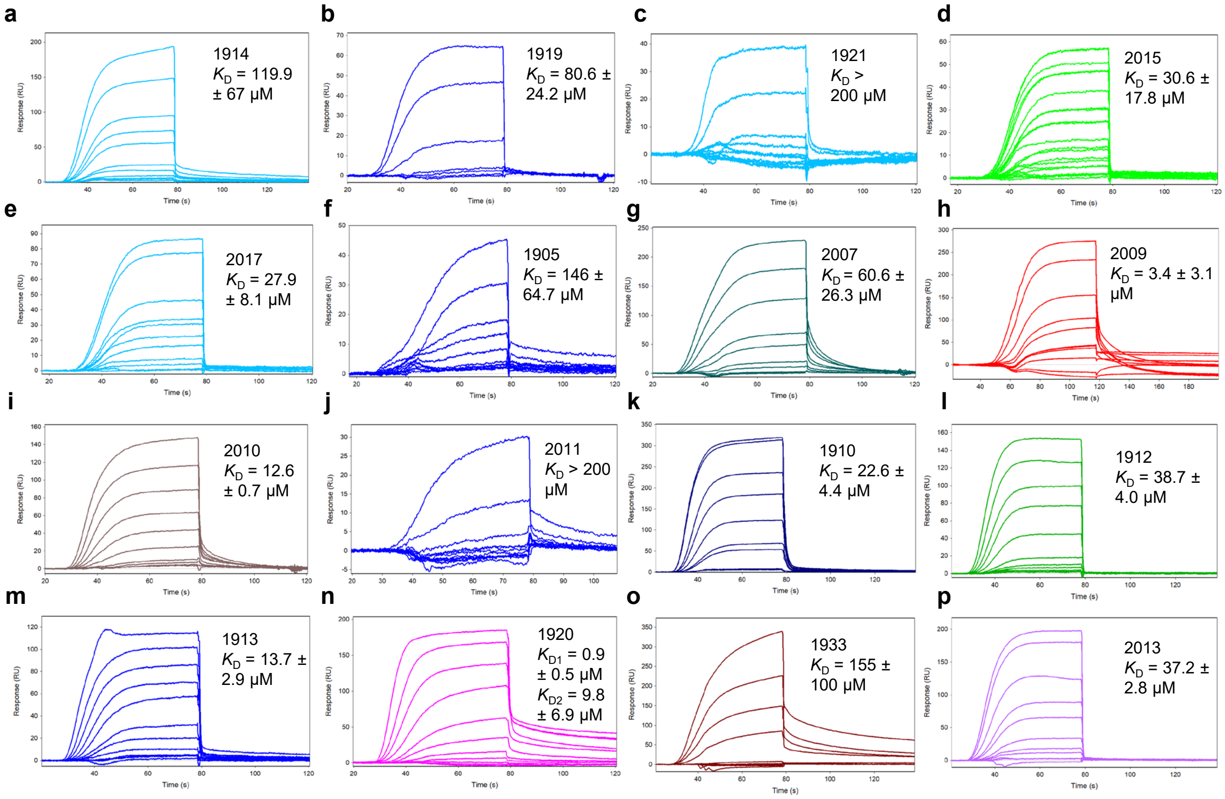

Supplementary Fig. 3. Surface plasmon resonance (SPR) studies characterize synthetic peptide binding to FAK focal adhesion targeting (FAT) domain. Representative SPR sensograms and average K_D ± standard deviation for peptides (**a**) 1914 (n=4), (**b**) 1919 (n=4), (**c**) 1921 (n=3), (**d**) 2015 (n=4), (**e**) 2017 (n=4), (**f**) 1905 (n= 4), (**g**) 2007 (n=4), (**h**) 2009 (n= 3), (**i**) 2010 (n=2), (**j**) 2011 (n= 3), (**k**) 1910 (n= 4), (**l**) 1912 (n=4), (**m**) 1913 (n= 4), (**n**) 1920 (n= 3), (**o**) 1933 (n= 3), (**p**) 2013 (n=3). All values for 'n' represent independent biological replicates. Source data are provided as a Source Data file.

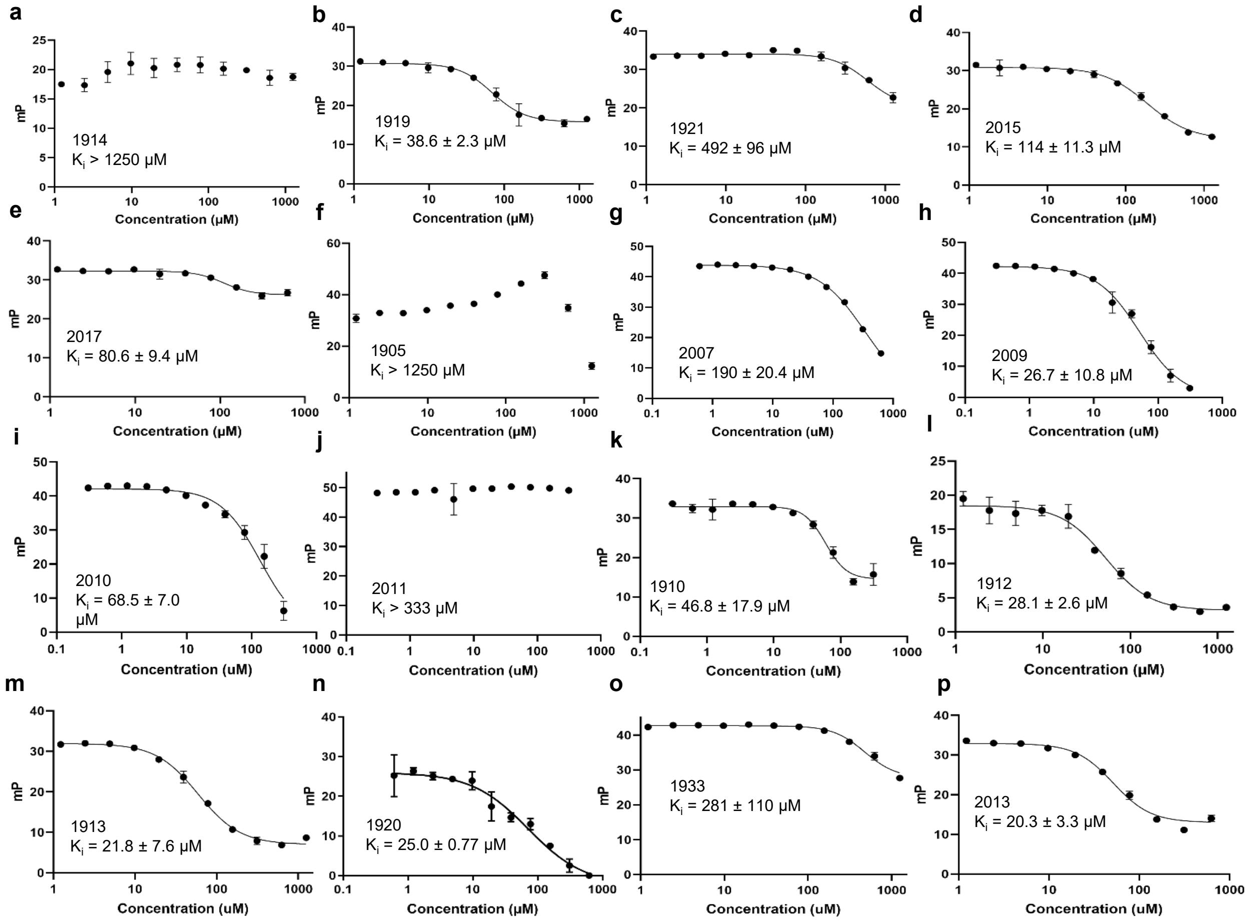

Supplementary Fig. 4. **Fluorescence Polarization (FP) assay characterizes peptide effect on inhibition of the FAK-paxillin interaction.** Representative FP competition curves and average K_i ± standard deviation for peptides: (**a**) 1914, (**b**) 1919, (**c**) 1921, (**d**) 2015, (**e**) 2017, (**f**) 1905, (**g**) 2007, (**h**) 2009, (**i**) 2010, (**j**) 2011, (**k**) 1910, (**l**) 1912, (**m**) 1913, (**n**) 1920, (**o**) 1933, (**p**) 2013. All averages are reported from 3 replicates (n=3). All values for 'n' represent independent biological replicates. Source data are provided as a Source Data file.

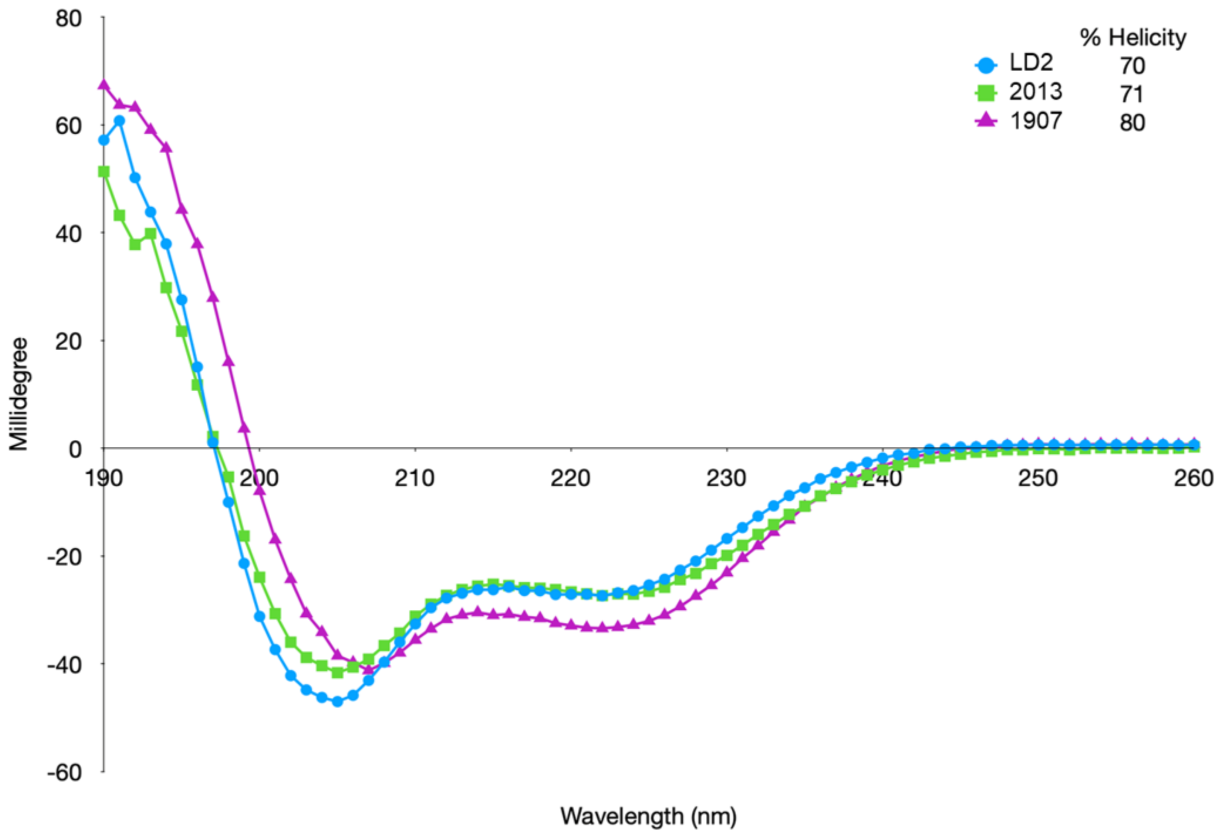

Supplementary Fig. 5. Hydrocarbon stapling enhances α-helicity of peptides. Comparative circular dichroism analysis of native LD2, stapled peptide 1907, and unstapled peptide 1907 (2013) demonstrate that hydrocarbon stapling can increase α-helical content by 10 % in aqueous sodium phosphate solution (pH 7.0). One biological replicate (n=1) was performed. Source data provided as a Source Data file.

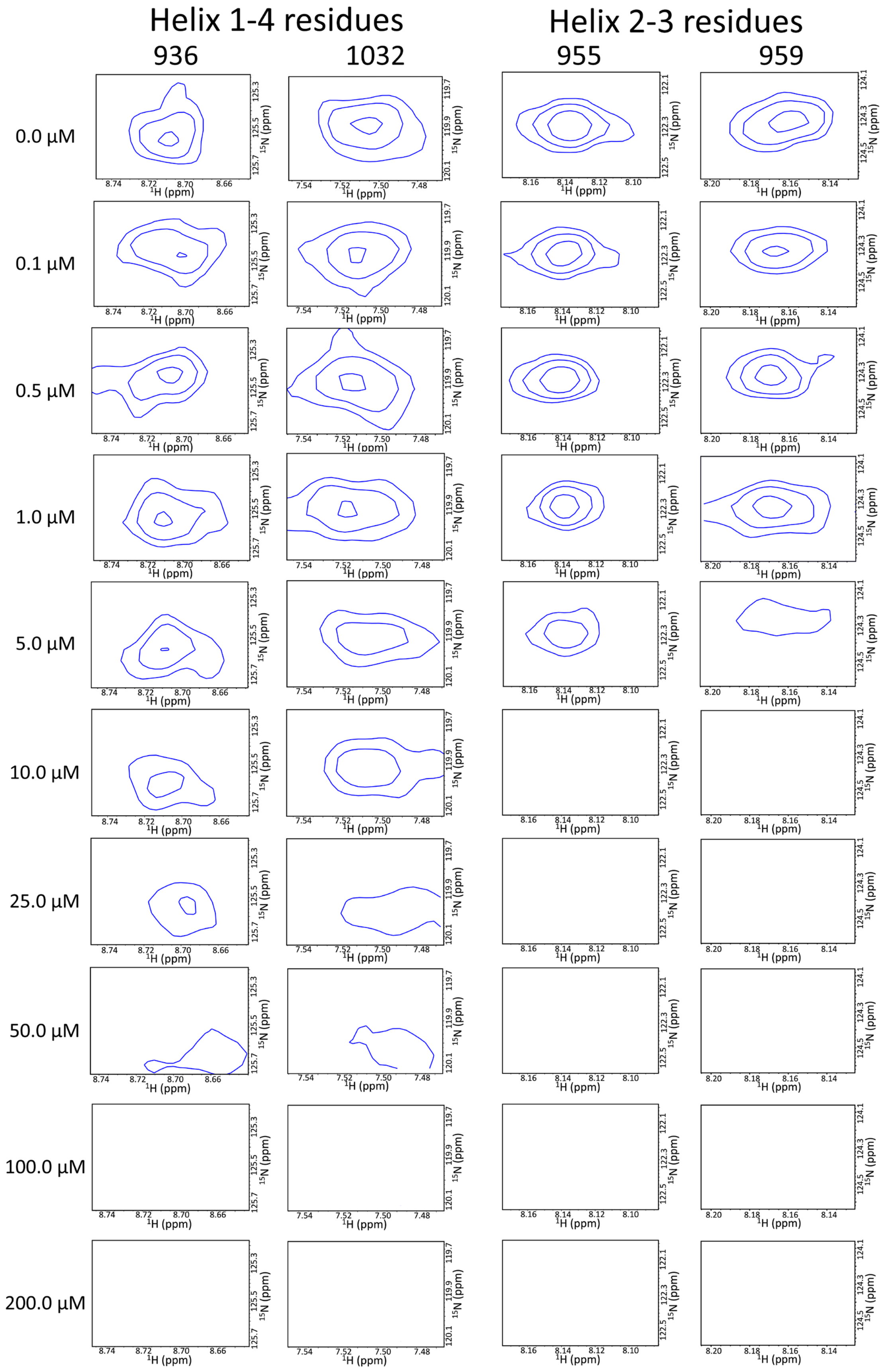

Helix 1-4 residues
Helix 2-3 residues
936
1032
955
959
0.0 µM
0.1 µM
0.5 µM
1.0 µM
5.0 µM
10.0 µM
25.0 µM
50.0 µM
100.0 µM
200.0 µM
^{1}H (ppm)
^{15}N (ppm)
8.74 8.72 8.70 8.66
125.3 125.5 125.7
7.54 7.52 7.50 7.48
119.7 119.9 120.1
8.16 8.14 8.12 8.10
122.1 122.3 122.5
8.20 8.18 8.16 8.14
124.1 124.3 124.5

Supplementary Fig. 6. HSQC-NMR peak integration studies identify the FAT helix 2-3 binding site as the higher affinity site for peptide 1907 compared to the helix 1-4 site. HSQC contour plots of two residues in the 1-4 domain (936 and 1032) and two residues in the 2-3 domain (955 and 959) at various concentrations of 1907 (200, 100, 50, 25, 10, 5, 1, 0.5, 0.1, and 0.0 μM) and 100 μM FAT demonstrate that saturation (loss of peak integration) is achieved in the 2-3 domain at lower concentrations than at the 1-4 binding site. Integration analysis of these peaks yield the binding curves shown in Figure 3.

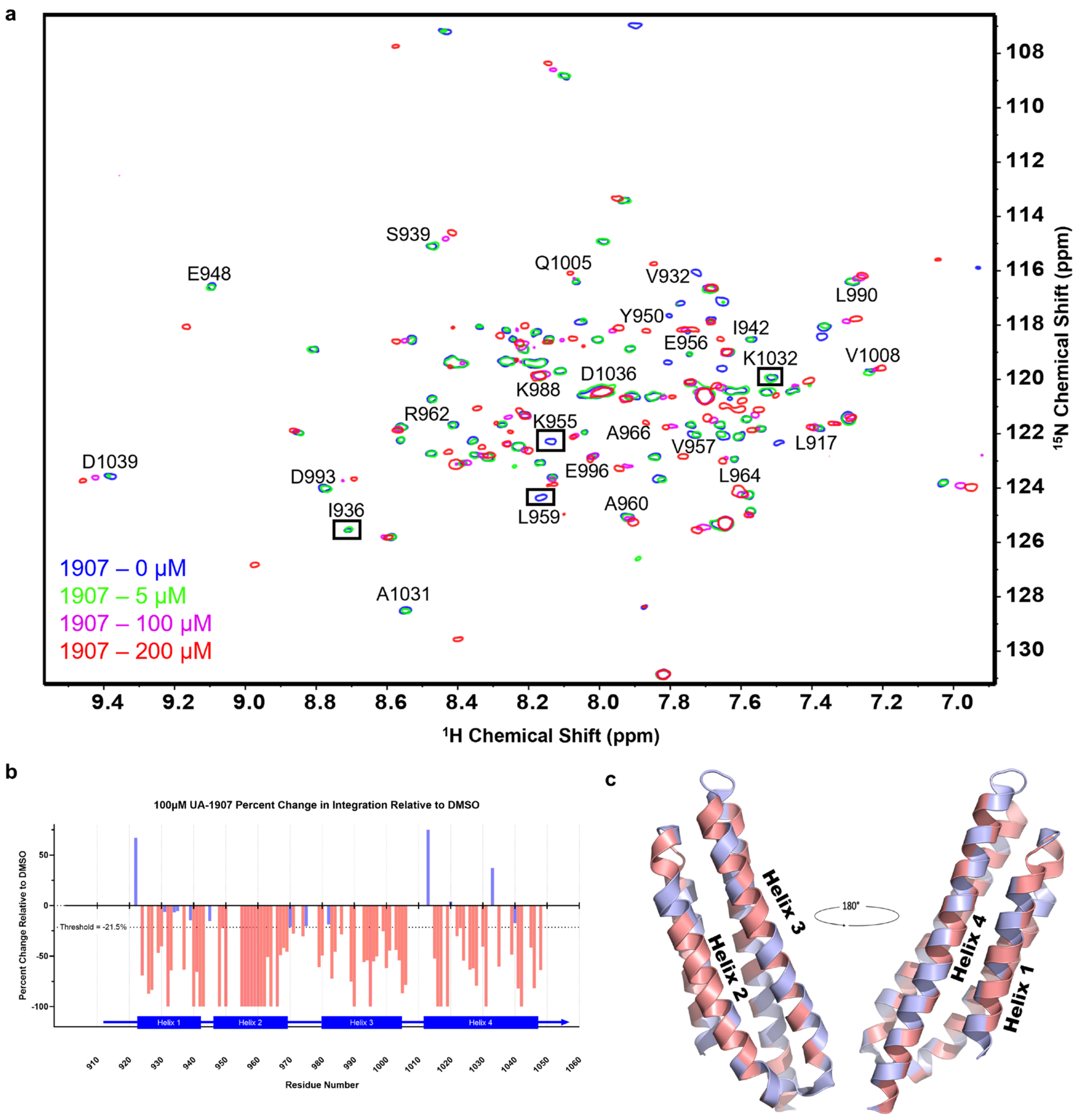

Supplementary Fig. 7. Stapled peptide 1907 displays binding at both FAT helix 2-3 and helix 1-4 sites at high concentrations. (**a**) Overlay of 2D HSQC spectra acquired with varying concentrations of 1907 and 100 μM FAT. Two-dimensional signals represented in red, magenta, green, and blue were acquired in the presence of 200 μM, 100 μM, 5 μM, and 0 μM of 1907 respectively. Residues that were previously shown to have notable chemical shift perturbations in the presence of LD2 and have notable differences in integration between high and low concentrations of 1907, are marked. (**b**) Histogram of the residue-wise HSQC peak volume change, shown as percent relative to DMSO upon incubation of FAT with

100 μM 1907. Highlighted residues match those in panel (**c**) Molecular model (PDB ID: 1OW8[24]) [https://doi.org/10.2210/pdb1OW8/pdb] of the 1907 binding surfaces on FAT. Residues with a ^{1}H-^{15}N HSQC peak volume decrease of more than 21% (as derived from 1 standard deviation in Figure 3) are highlighted in the color salmon. Source data for (b) are provided as a Source Data file.

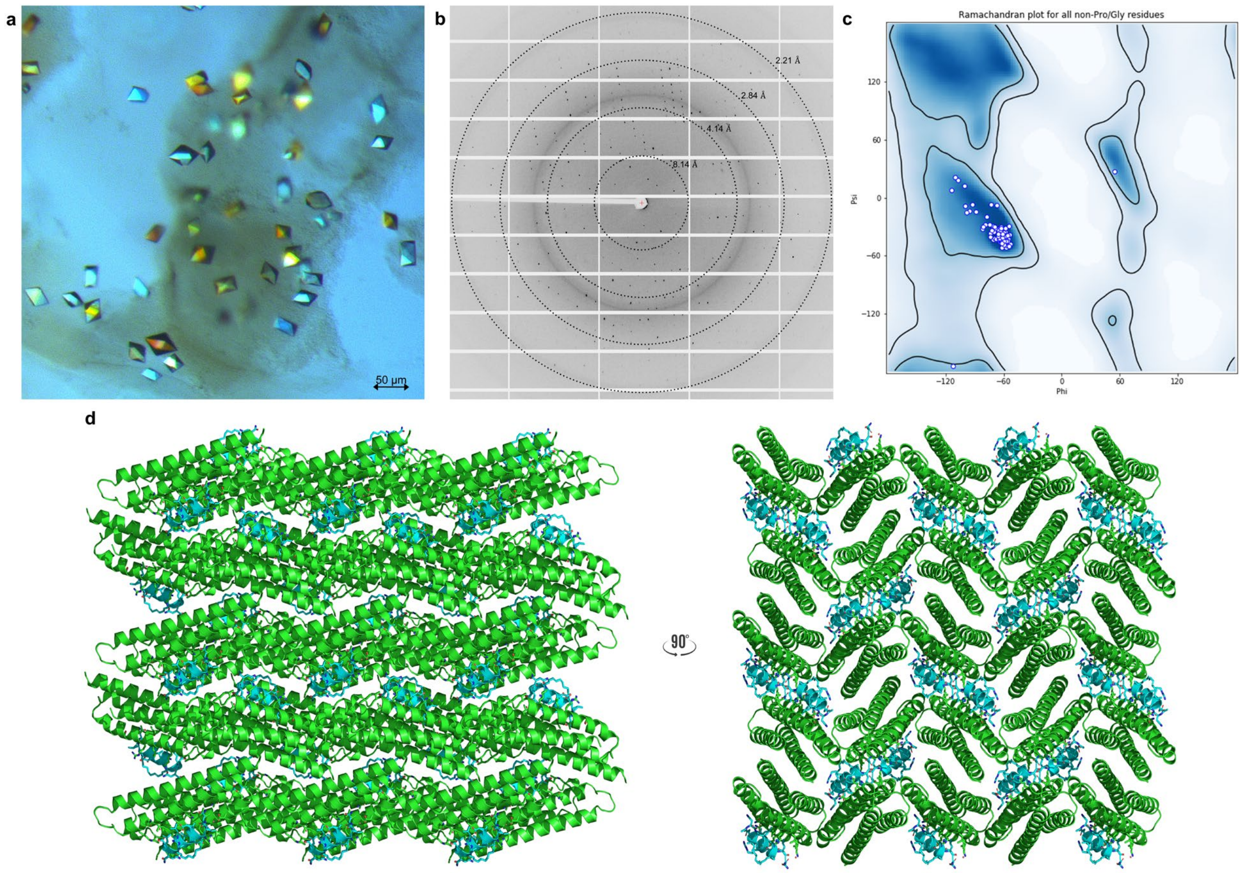

Supplementary Fig. 8. FAT-1907 X-ray co-crystallography experiments. (**a**) Co-crystallized FAT-1907 crystals used in X-ray data collection. (**b**) 2D X-ray diffraction pattern eliciting well-ordered protein crystal lattice. (**c**) Ramachandran plot after refinement indicating no structural outliers in the final model. (**d**) Crystal lattice of the solved structure belonging to space group P 2_1 2_1 2_1.

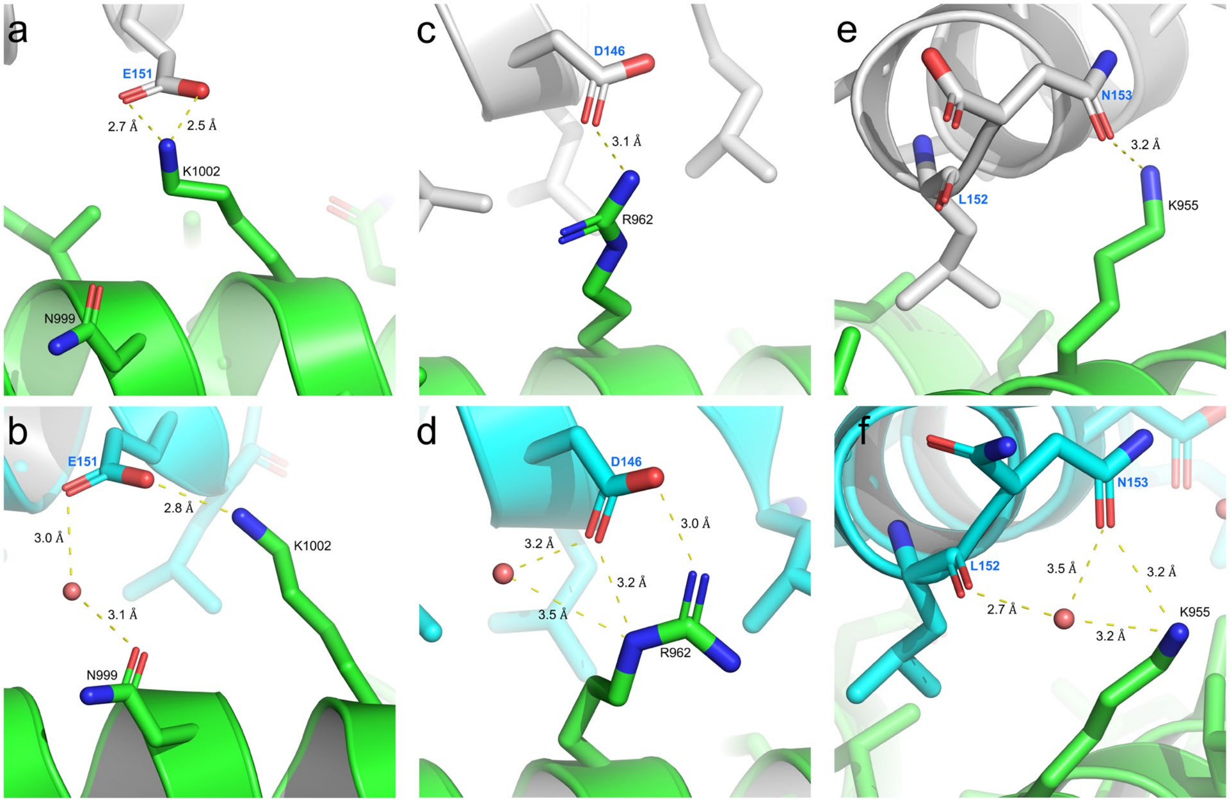

Supplementary Fig. 9. Conformational and interaction changes between native paxillin LD2 peptide and stapled peptide 1907 as observed in the X-ray co-crystal structure. Shifts in side chain orientation are seen in FAT (green) residues (**a**, **b**) K1002 and (**c**, **d**) R962 when bound by (**a**, **c**) LD2 (grey) compared to (**b**, **d**) 1907 (cyan) mediated by coordinated solvent molecules (salmon spheres). FAT residue (**e**, **f**) K955 show minimal perturbations in the (**e**) LD2 model compared to (**f**) 1907, with the addition of a coordinated solvent molecule forming a new interaction with 1907 residue L152 (LD2 PDB ID 1OW8 [https://doi.org/10.2210/pdb1OW8/pdb]; 1907 PDB ID 6PW8 [https://doi.org/10.2210/pdb6PW8/pdb]).

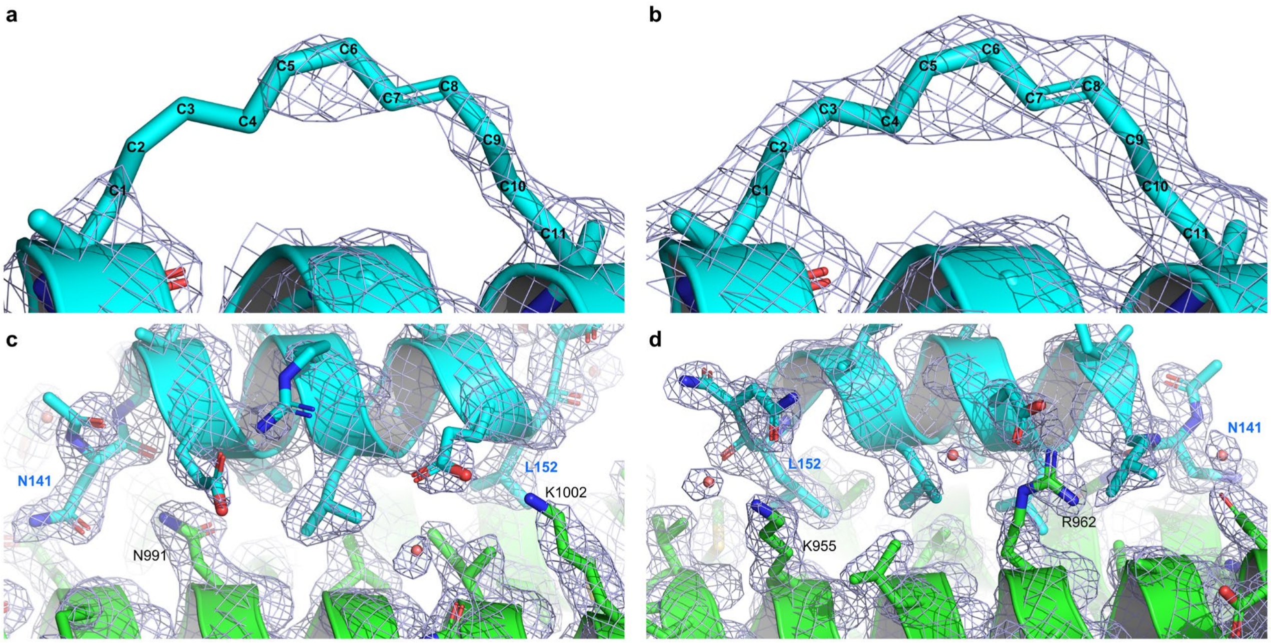

Supplementary Fig. 10. Electron density maps of FAT-1907 X-ray co-crystal structure. Modeling of peptide 1907 hydrocarbon-staple to electron density map at a contour level of (**a**) 0.85 sigma and (**b**) 0.25 sigma. Electron density map of the (**c**) anterior and (**d**) posterior sides of the FAT-1907 protein-peptide interface at 0.85 sigma.

a

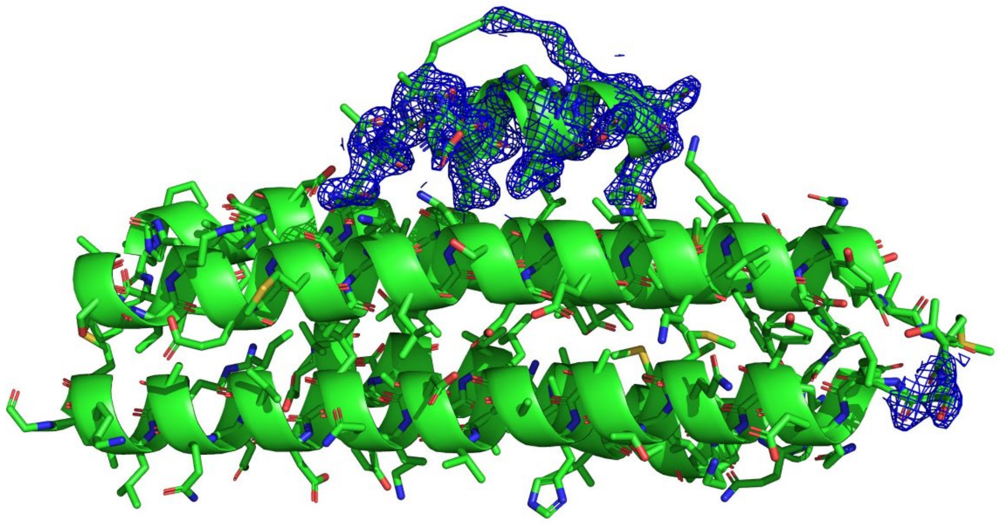

b

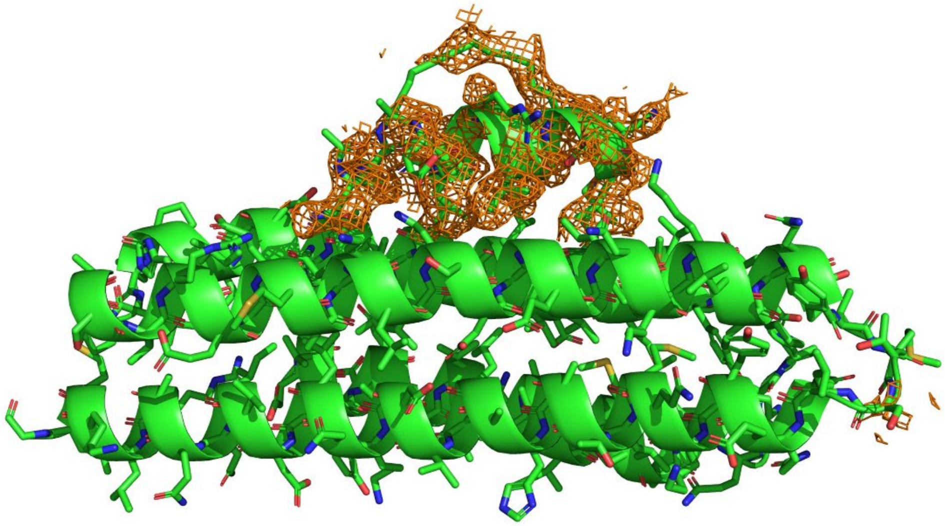

Supplementary Fig. 11. Additional electron density maps of FAT-1907 X-ray co-crystal structure. (**a**) 2Fo-Fc electron density for chain B is at sigma level of 1.0 depicted (in blue). (**b**) Polder map is depicted with a contour level of 1.0 sigma (in orange).

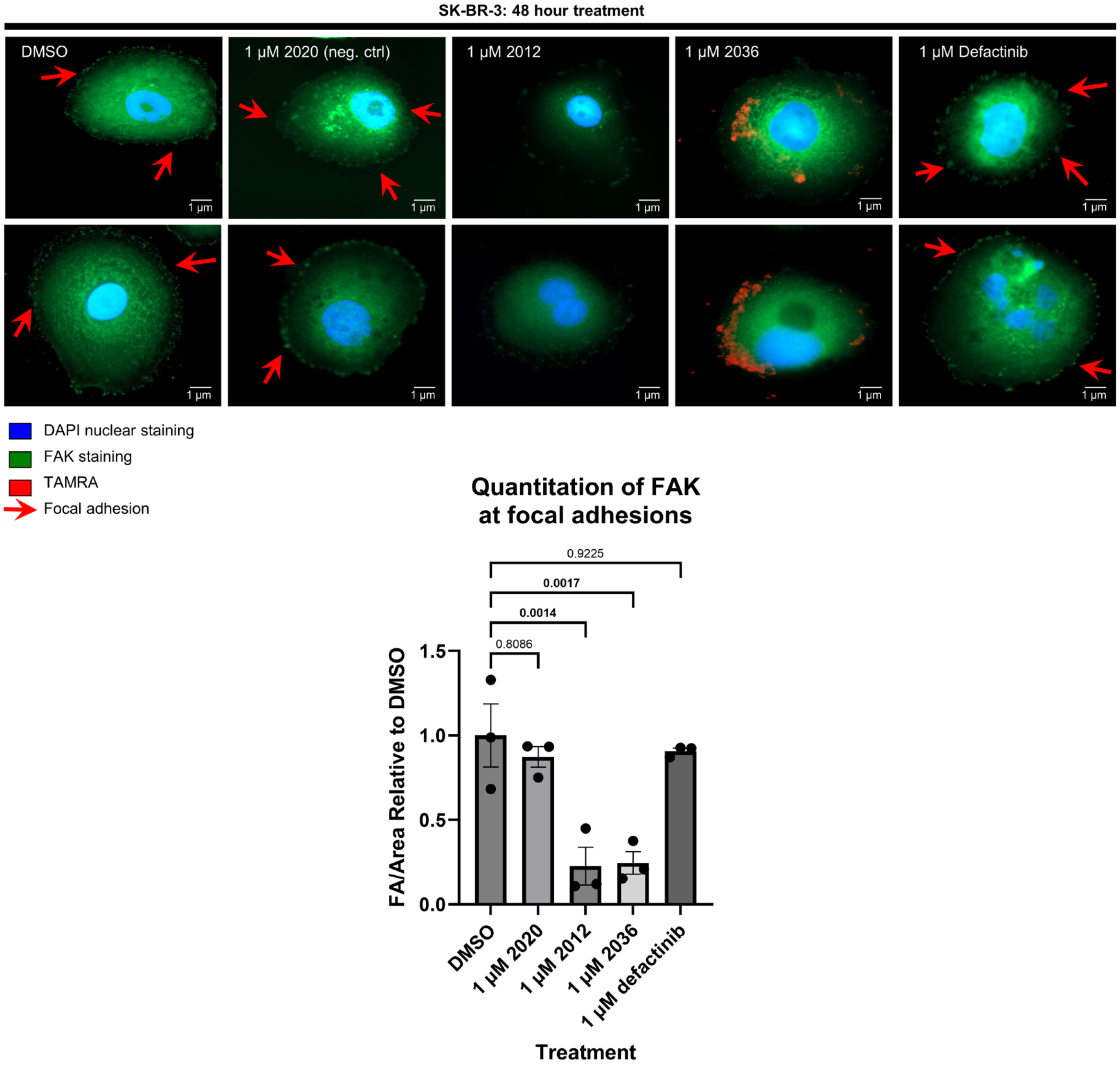

Supplementary Fig. 12. Immunofluorescence assay showing 2012 cell permeability and de-localization of FAK from focal adhesions. Tests were performed in SK-BR-3 breast cancer cells with 1 μM treatment of 2020 (neg. control), 2012, 2036 (TAMRA-tagged 2012), defactinib, or vehicle (DMSO) control. Green focal adhesion foci are indicated with red arrows. Intracellular localization of 2012 was visualized using peptide 2036 (TAMRA-tagged 2012). Quantitation of focal adhesions in bar graph obtained via ImageJ analysis, bar graph made using GraphPad Prism showing mean ± SD and corrected p-values. Bolded p-values indicate statistically significant values where $p<0.05$. Three independent biological replicates (n=3) were performed. One-way ANOVA statistical analysis and Dunnett's multiple comparisons test performed.

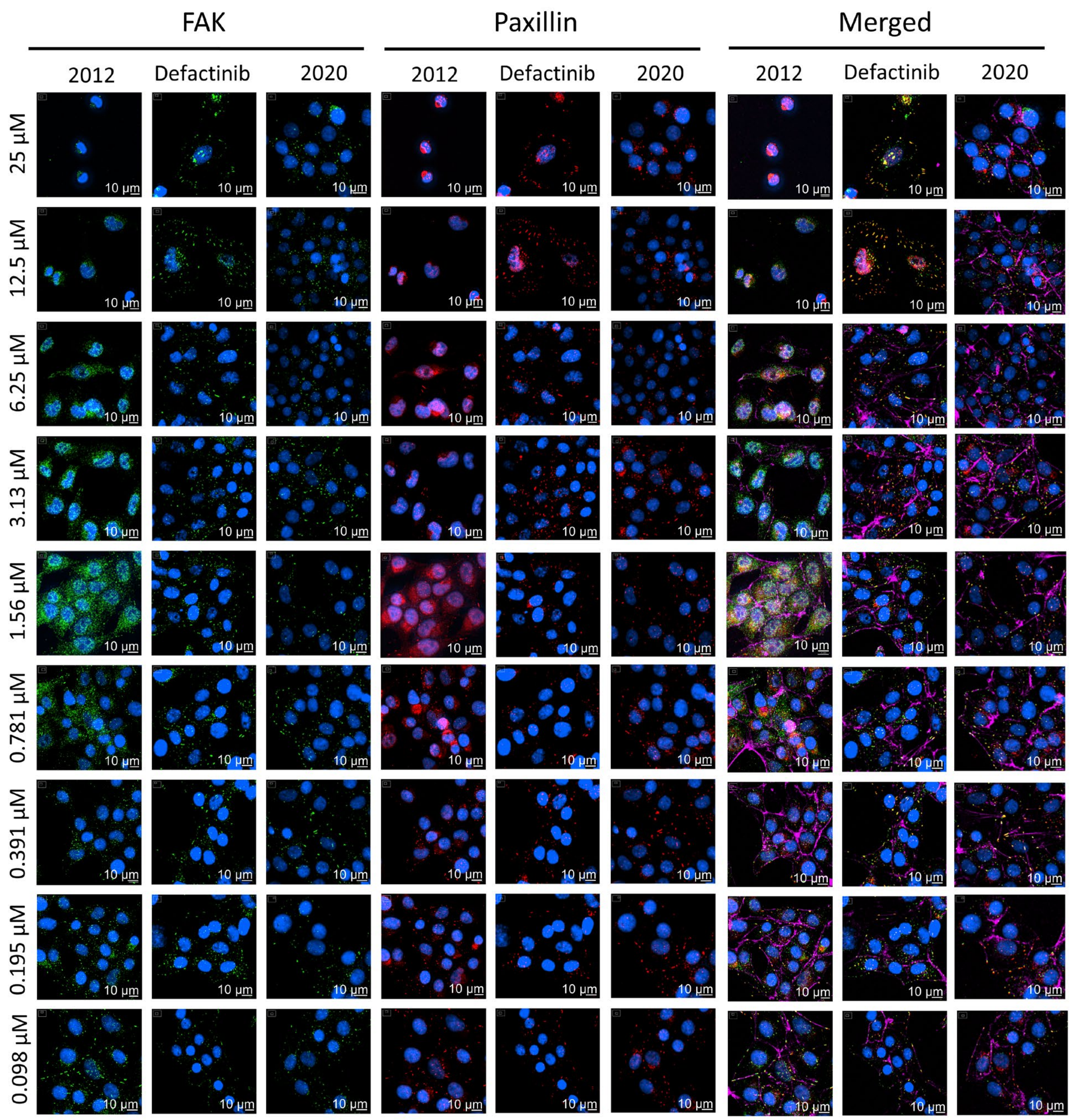

Supplementary Fig. 13. Immunofluorescence dose response assay showing 2012 induced de-localization of FAK from focal adhesions in SK-MEL-147 cells. Immunofluorescence staining of FAK (green), Paxillin (red), and DAPI (blue) in SK-MEL-147 melanoma cells after 48 hours of dose-response treatment 2012, defactinib, and 2020 (neg. control).

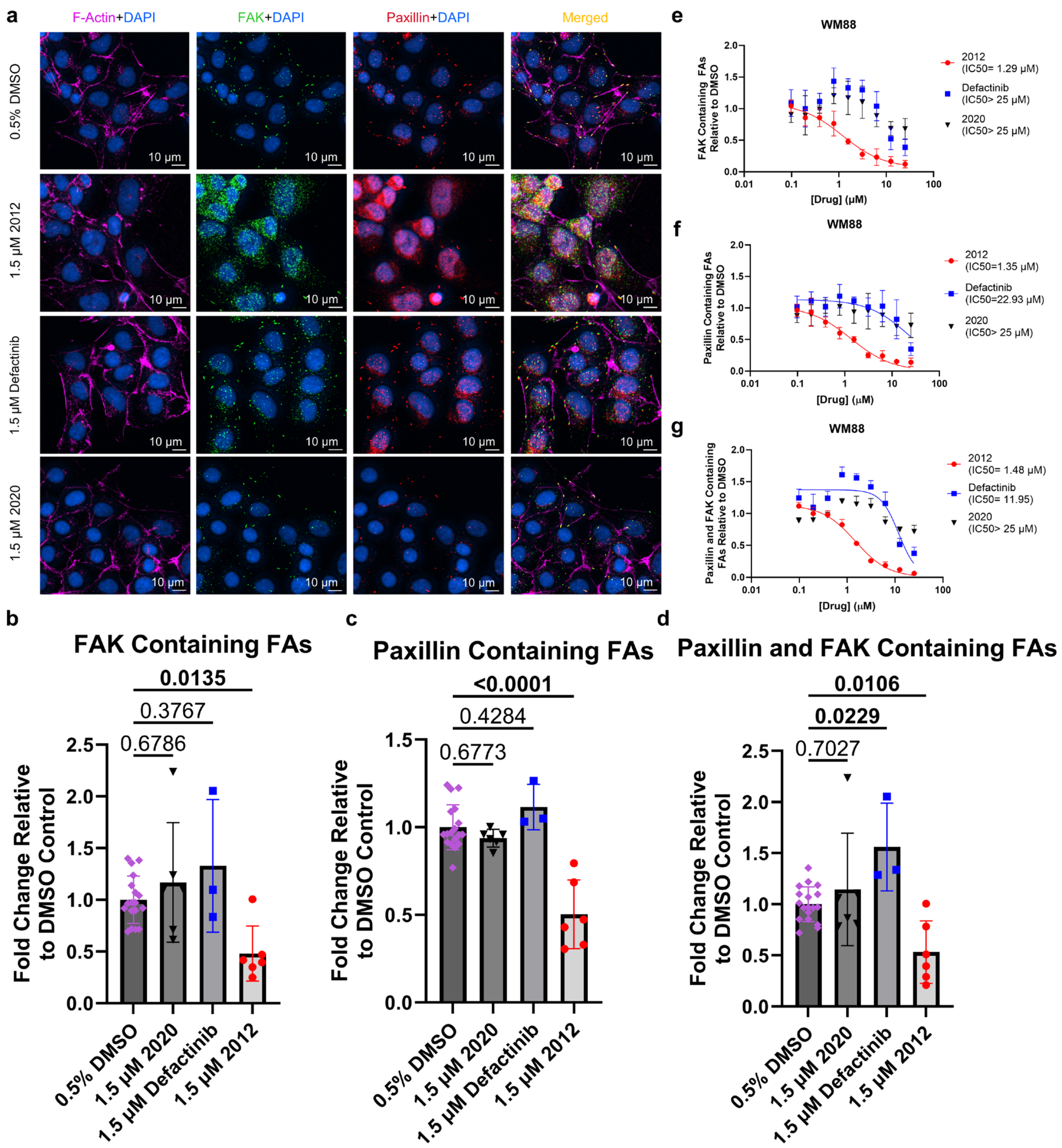

Supplementary Fig 14. Immunofluorescence assay showing 2012 induced de-localization of FAK from focal adhesions in WM88 melanoma cells. (**a**) Immunofluorescence staining of F-actin (magenta), FAK (green), Paxillin (red), and DAPI (blue) in WM88 melanoma cells after 48 hours of treatment with 1.5 µM 2012, defactinib, and 2020 (neg. control) along with 0.5% DMSO (vehicle control) and corresponding changes in (**b**) FAK, (**c**) paxillin, and (**d**) dual FAK-paxillin containing focal adhesions. One-way ANOVA analyses and Dunnett's multiple comparison tests were performed in (b-d) and changes in

FA composition denoted with corrected p-values provided. Bolded p-values indicate statistically significant values where $p<0.05$. Sample size for (b-d) was 18 (n=18) for DMSO (vehicle control); 6 (n=6) for 2012 and 2020 treated groups; and 3 (n=3) for defactinib treated groups. All values for ‘n’ represent independent biological replicates. Dose response curves showing the mean ± SD reduction of (**e**) FAK, (**f**) paxillin, or (**g**) dual FAK-paxillin containing focal adhesions and corresponding IC_{50} values after 48 hours of treatment with 2012, defactinib, and 2020 relative to 0.5% DMSO control in WM88 cells. Three biological replicates were performed (n=3). All source data are provided as a Source Data file.

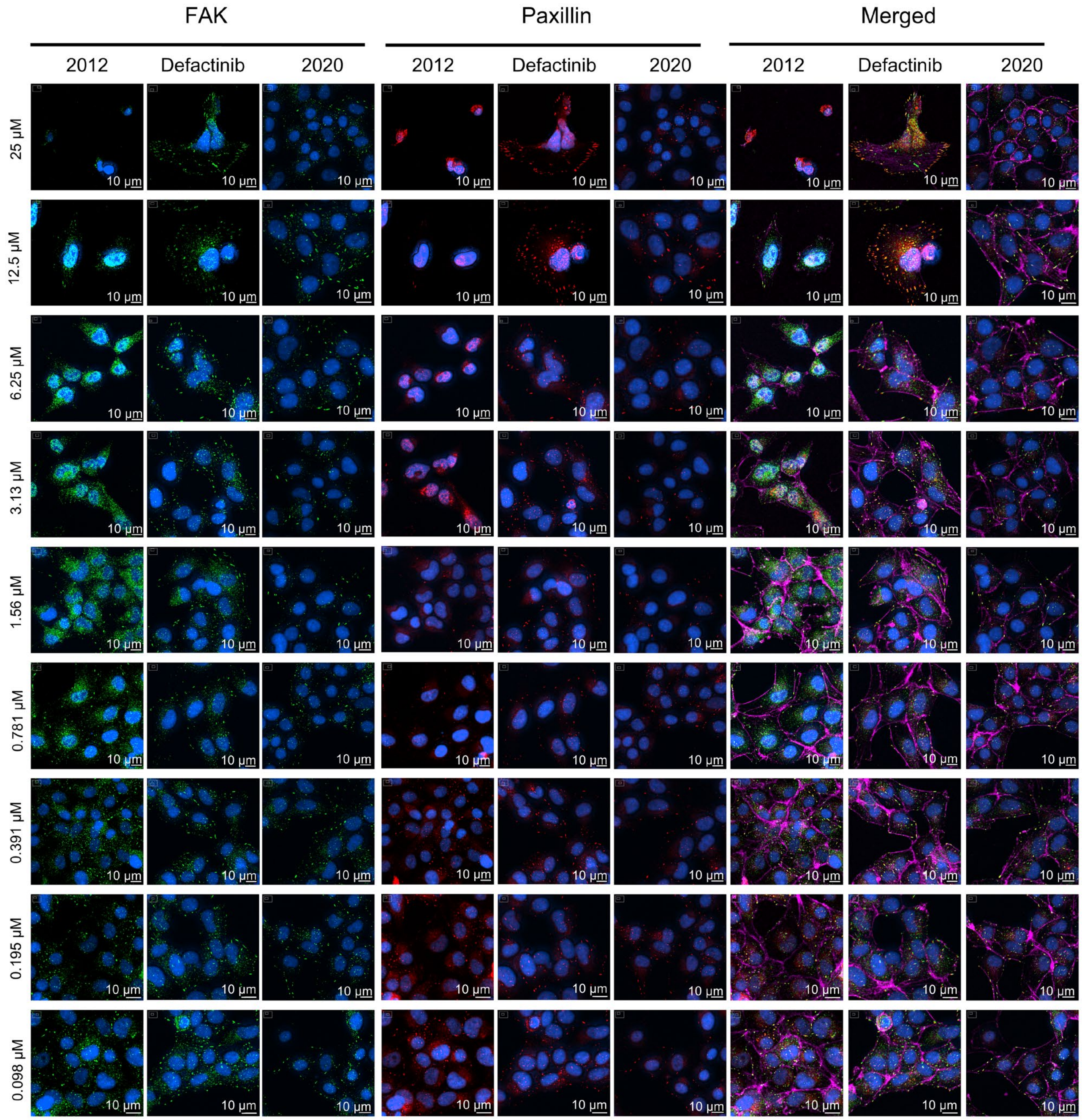

Supplementary Fig 15. Immunofluorescence dose response assay showing 2012 induced de-localization of FAK from focal adhesions in WM88 melanoma cells. Immunofluorescence staining of FAK (green), Paxillin (red), and DAPI (blue) in WM88 melanoma cells after 48 hours of dose-response treatment 2012, defactinib, and 2020 (neg. control).

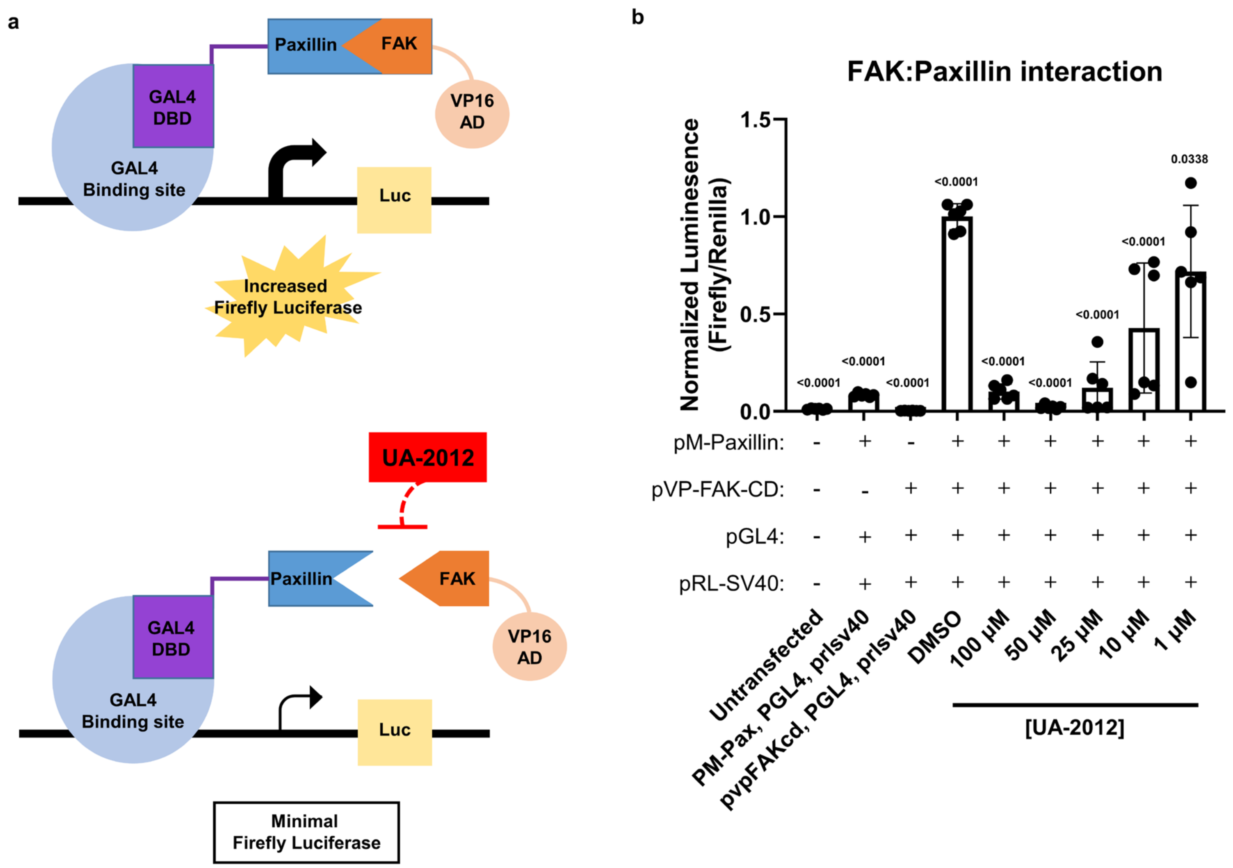

Supplementary Fig. 16. Interaction between FAK and paxillin proteins is disrupted by 2012 treatment. HEK293T cells, treated with 2012 (2 hour pre-treatment followed by 24 hour post-treatment), were transfected with plasmid fusion constructs for 24 hours in a mammalian 2-hybrid system to measure critical FAT-paxillin protein-protein interactions. (**a**) Graphical representation of the FAK-paxillin Mammalian 2-Hybrid System. (**b**) Mean ± SD luminescence of various 2012 treatments compared to DMSO-vehicle control and single plasmid-transfection controls from 6 biological replicates (n= 6). Data are normalized for transfection efficiency via Renilla luciferase expression. One-way ANOVA statistical analysis and Dunnett's multiple comparisons test performed and corrected p-values reported. Bolded p-values indicate statistically significant values where p<0.05. Source data provided as Source Data file.

a

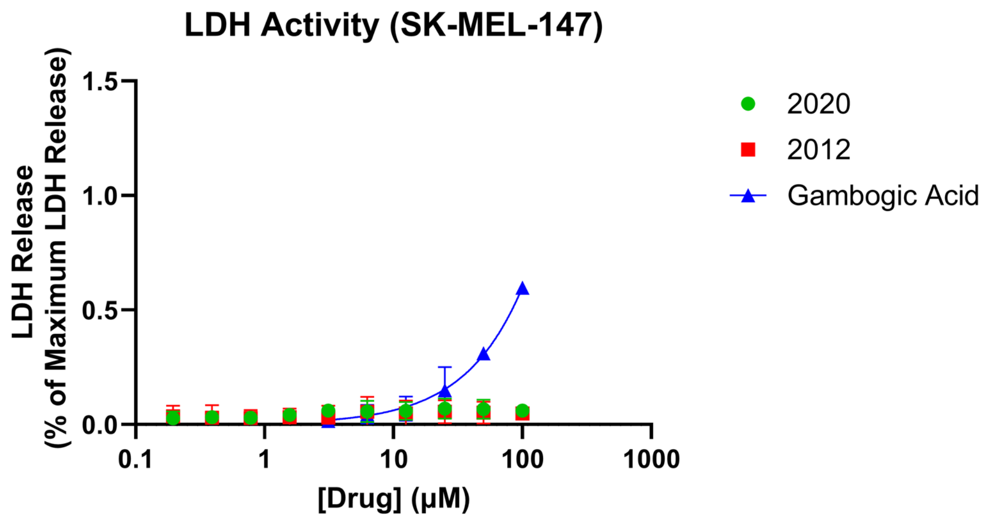

b

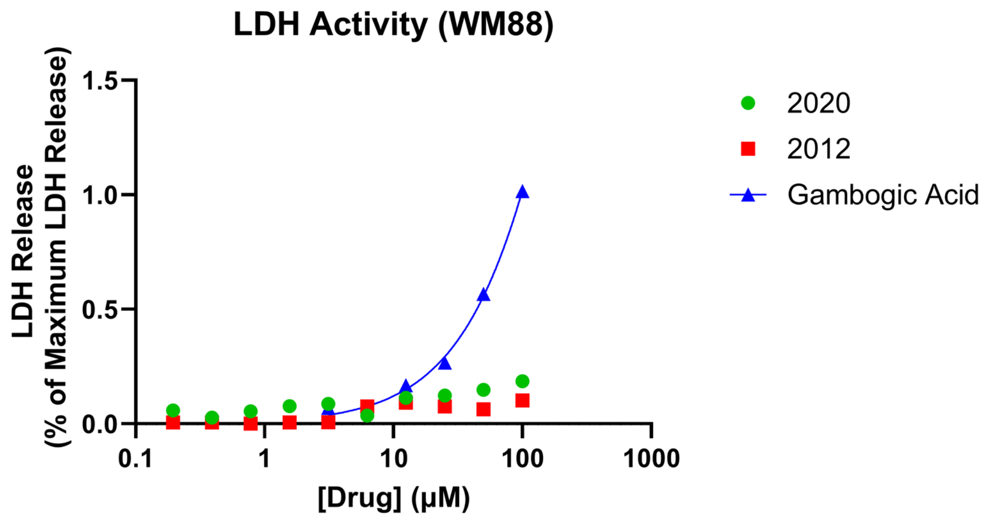

Supplementary Fig 17. LDH release assay shows even at high micromolar doses of peptide 2012 and 2020 do not result in damage to plasma membrane. LDH release assay preformed in human melanoma cell lines (**a**) SK-MEL-147 and (**b**) WM88 using compounds 2012, 2020 (neg. control), and Gambogic Acid (pos. control) at titrating concentrations ranging from 100 μM to 200 nM for 45 minutes. Dose response curves generated in GraphPad Prism and report mean ± SD LDH Release relative to the maximum possible LDH release of three biological replicates (n=3). Source data are provided as Source Data file.

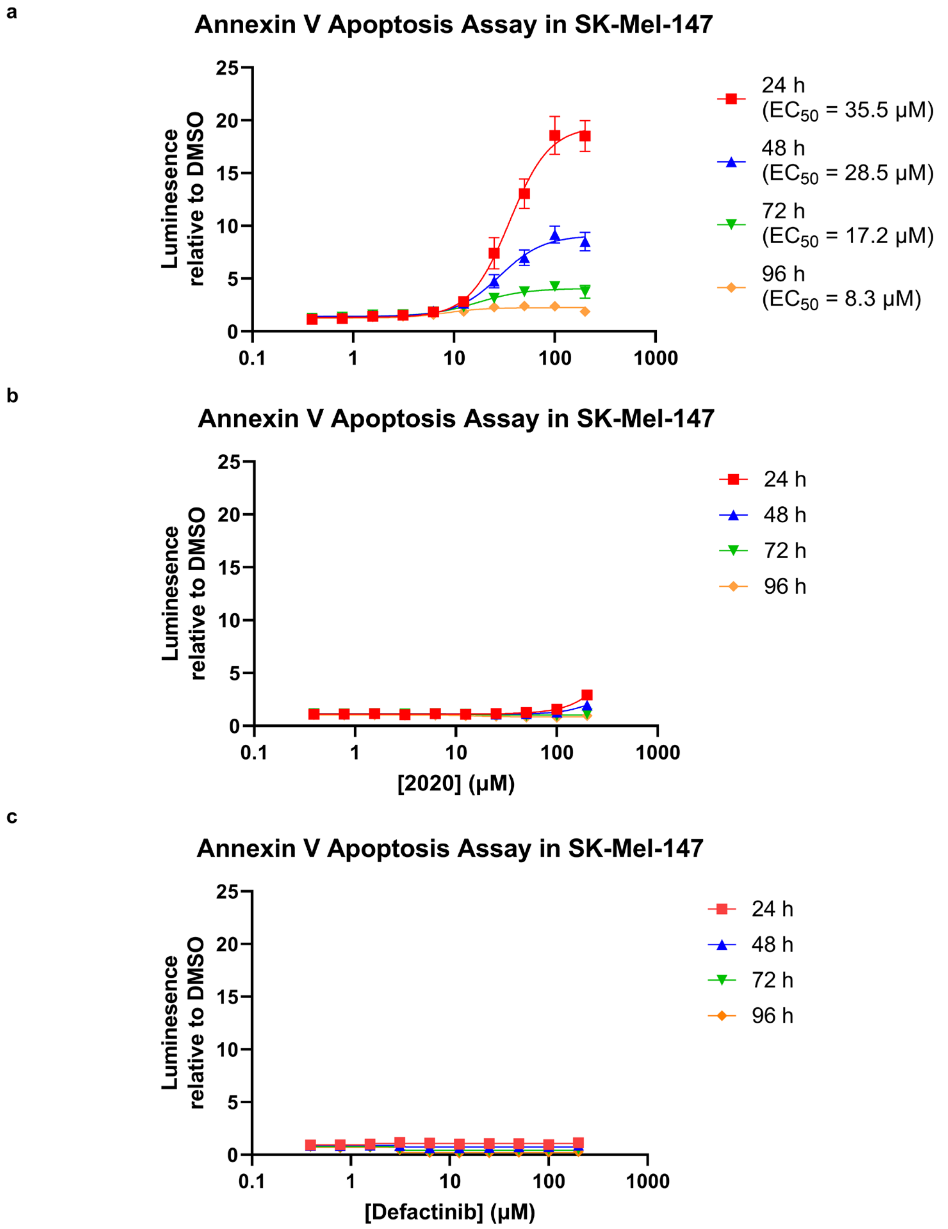

Supplementary Fig. 18. Peptide 2012 induces apoptosis in dose-dependent and time-dependent manner via Annexin V apoptosis time-course study. SK-MEL-147 cells treated with (**a**) 2012, (**b**) 2020, and (**c**) defactinib are evaluated for apoptosis using RealTime-Glo system for Annexin V binding. EC_{50} curves representing data obtained every 24 hours, over the course of 96 hours total. EC_{50} curves fit using GraphPad Prism reporting mean ± SD of (a) 7 biological replicates (n=7); (b) 5 biological replicates (n =5); and (c) 5 biological replicates (n =5). Source data are provided as Source Data files.

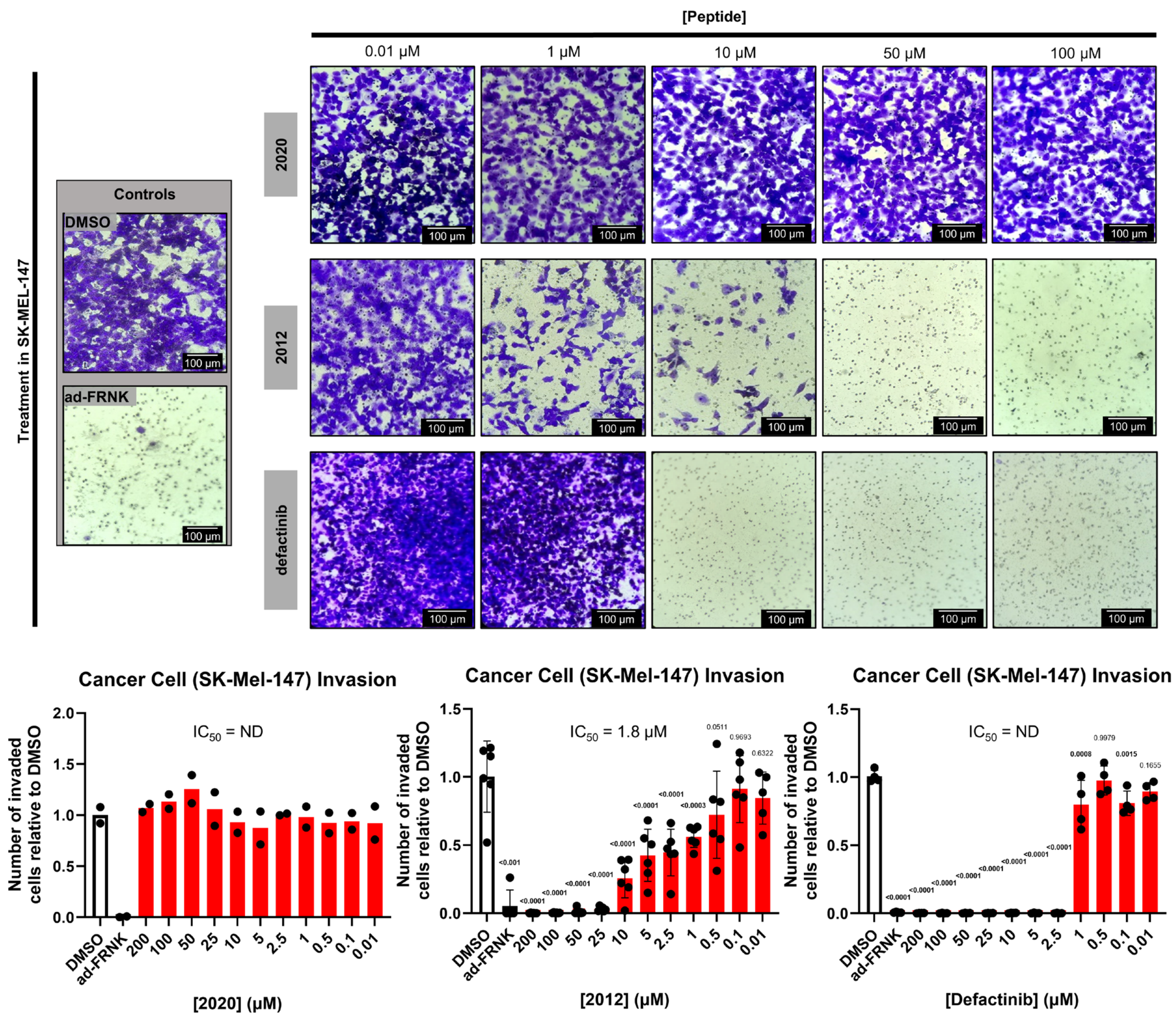

Supplementary Fig. 19. Peptide 2012 causes dose-dependent reduction in cancer-cell invasion via Transwell invasion assay. Representative brightfield images of invaded SK-MEL-147 cells on transwell inserts at 10X magnification following peptide treatment. Images show invaded cells after treatment with various concentrations of 2020 (top panel), 2012 (middle panel), and defactinib (bottom panel), in addition to DMSO vehicle-control (left, top panel) and ad-FRNK positive control (left, bottom panel). Bar graphs reveal mean number of invaded cells ± SD and calculated IC_{50} values across all doses of 2020 (left graph, n=2), 2012 (middle graph, n=6), and defactinib (right graph, n=4). All values for 'n' represent independent biological replicates. One-way ANOVA analyses and Dunnet's multiple comparison tests were performed

for 2012 and defactinib treated groups and corrected p-values are reported. Bolded p-values indicate statistically significant values where $p<0.05$. All source data are provided as Source Data files.

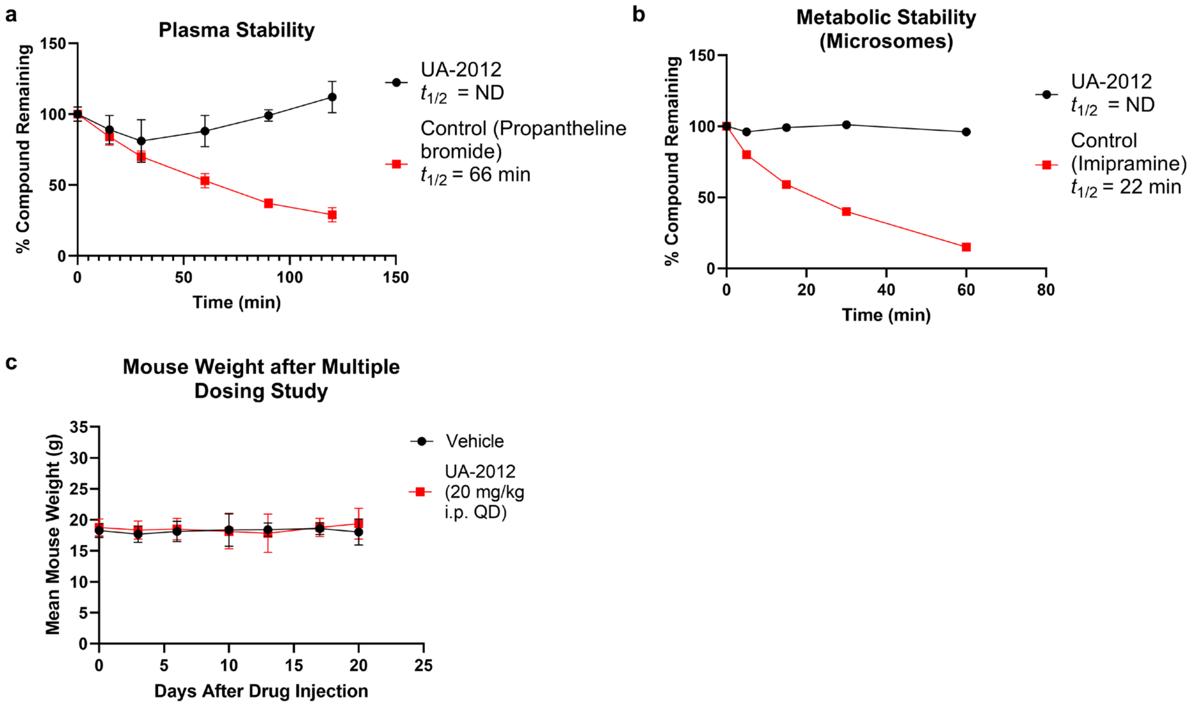

Supplementary Fig. 20. Peptide 2012 shows promising plasma and metabolic stability and no signs of toxicity *in vivo*. Additional DMPK assays demonstrate: (**a**) plasma stability of 2012 in mouse plasma compared to control molecule, propantheline bromide (n=3), and (**b**) microsomal stability of 2012 and control molecule, imipramine, in mouse liver microsomes (n=2). (**c**) Curves obtained from B16F10 syngeneic mouse model of melanoma indicating mean mouse body weight ± SD (n = 8) during 2012 and vehicle-control treatments, a measure of possible toxicity and tolerance to treatments. All values for ‘n’ represent independent biological replicates. Source data are provided as Source Data files.

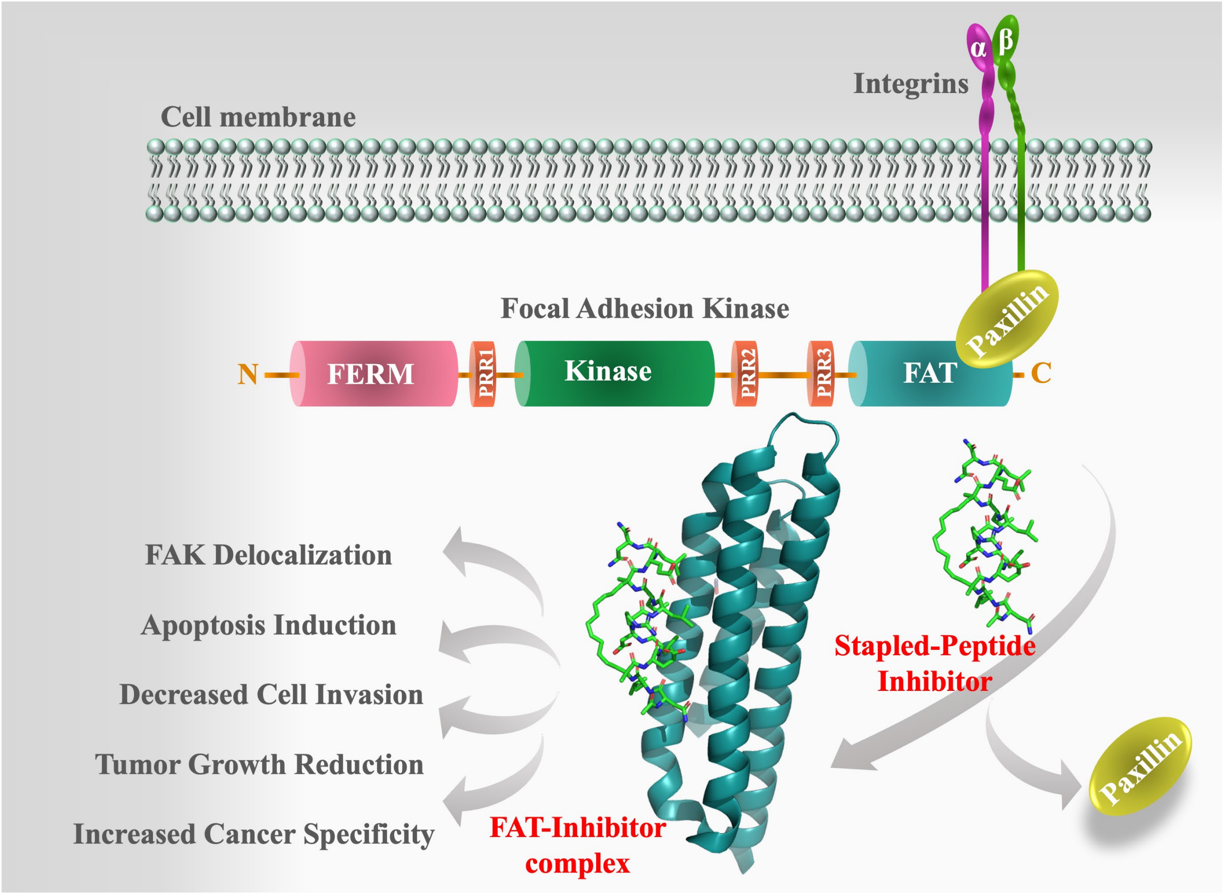

Supplementary Fig. 21. Summary Figure: discovery of hydrocarbon-stapled paxillin mimetics with improved anti-cancer efficacy and selectivity compared to first-generation kinase-domain inhibitors. Herein, we describe the structure-based optimization and synthesis of a series of hydrocarbon-stapled paxillin mimetics. We report the X-ray co-crystal structure of a FAK-scaffold inhibitor bound to its cognate target domain (FAT domain, PDB 6PW8). These peptides displayed anti-cancer biology not previously observed with first-generation FAK-kinase domain inhibitors, including FAK delocalization from focal adhesions, induction of apoptosis, dose-dependent inhibition of invasion, and high cancer: normal cell selectivity.

ID	Synonyms	Exact mass	Mass obs	Mass theor	Mass assign	Mass method
LD2	Ac-NLSELDRLLLELN-NH_2	1581.87	792.1	791.94	1/2*[M+2H]$^{2+}$	ESI+
1907	Ac-NL$\mathbf{R_8}$ELDRLL$\mathbf{S_5}$ELN-NH_2	1673.97	838.1	837.99	1/2*[M+2H]$^{2+}$	ESI+
1914	Ac-NLSE$\mathbf{R_8}$DRLLLE$\mathbf{S_5}$N-NH_2	1647.92	825.1	824.97	1/2*[M+2H]$^{2+}$	ESI+
1919	Ac-NLSE$\mathbf{S_5}$DRL$\mathbf{S_5}$LELN-NH_2	1605.87	804.0	803.94	1/2*[M+2H]$^{2+}$	ESI+
1921	Ac-NLSELDRL$\mathbf{R_5}$LE$\mathbf{S_5}$N-NH_2	1605.87	804.0	803.94	1/2*[M+2H]$^{2+}$	ESI+
2015	Ac-NLSELDR$\mathbf{S_5}$LLE$\mathbf{S_5}$N-NH_2	1605.87	804.1	803.94	1/2*[M+2H]$^{2+}$	ESI+
1925	Ac-NLSE$\mathbf{R_8}$DRAALE$\mathbf{S_5}$N-NH_2	1563.83	783.0	782.92	1/2*[M+2H]$^{2+}$	ESI+
1931	Ac-NLRE$\mathbf{S_5}$DRL$\mathbf{S_5}$RELN-NH_2	1717.96	860.5	859.99	1/2*[M+2H]$^{2+}$	ESI+
1905	Ac-NLS$\mathbf{R_8}$LDRLLL$\mathbf{S_5}$LN-NH_2	1616.00	809.1	809.01	1/2*[M+2H]$^{2+}$	ESI+
2017	Ac-N$\mathbf{R_8}$SELDRL$\mathbf{S_5}$LELN-NH_2	1648.90	825.1	825.46	1/2*[M+2H]$^{2+}$	ESI+
1906	H-NLS$\mathbf{R_8}$LDRLLN$\mathbf{S_5}$LN-NH_2	1574.95	788.6	788.48	1/2*[M+2H]$^{2+}$	ESI+
1910	Ac-NL$\mathbf{R_8}$QLDRLL$\mathbf{S_5}$QLN-NH_2	1672.00	848.1	848.00	1/2*[M+H+Na]$^{2+}$	ESI+
1912	Ac-NLAibELDRLLAibELN-NH_2	1551.86	777.1	776.94	1/2*[M+2H]$^{2+}$	ESI+
1913	Ac-DL$\mathbf{R_8}$ELDRLL$\mathbf{S_5}$ELD-NH_2	1675.94	839.5	838.98	1/2*[M+2H]$^{2+}$	ESI+
1920	Ac-SLGSNL$\mathbf{R_8}$ELDRLL$\mathbf{S_5}$ELNAVQH-NH_2	2453.36	1227.9	1227.69	1/2*[M+2H]$^{2+}$	ESI+
1928	Ac-RRNL$\mathbf{R_8}$ELDRLL$\mathbf{S_5}$ELNRR-NH_2	2298.38	2301.9	2299.38	[M+H]$^{+}$	MALDI
1933	Ac-NL$\mathbf{R_8}$EWDRLL$\mathbf{S_5}$EWN-NH_2	1819.96	911.1	910.99	1/2*[M+2H]$^{2+}$	ESI+
2007	Ac-NL$\mathbf{R_8}$ELDRLW$\mathbf{S_5}$ELN-NH_2	1746.97	874.6	874.49	1/2*[M+2H]$^{2+}$	ESI+
2009	Ac-NL$\mathbf{R_8}$ALDRLL$\mathbf{S_5}$ELN-NH_2	1615.97	809.1	808.99	1/2*[M+2H]$^{2+}$	ESI+
2010	Ac-NL$\mathbf{R_8}$ALDALL$\mathbf{S_5}$ELN-NH_2	1530.90	766.6	766.46	1/2*[M+2H]$^{2+}$	ESI+
2011	Ac-$\mathbf{R_8}$ELDRLL$\mathbf{S_5}$-NH_2	1090.68	1093.5	1091.68	[M+H]$^{+}$	MALDI
2013	Ac-NL$\mathbf{R_8}$*ELDRLL$\mathbf{S_5}$*ELN-NH_2 (unstapled)	1702.00	852.1	852.01	1/2*[M+2H]$^{2+}$	ESI+
2014	Ac-NL$\mathbf{R_8}$EEDRLL$\mathbf{S_5}$EEN-NH_2	1705.89	854.1	853.95	1/2*[M+2H]$^{2+}$	ESI+
2016	RhoB-NL$\mathbf{R_8}$ELDRLL$\mathbf{S_5}$ELN-NH_2	2128.22	710.3	710.41	1/3*[M+3H]$^{3+}$	ESI+
1917	Ac-SATRE$\mathbf{S_5}$DEL$\mathbf{S_5}$ASLSD-NH_2	1683.83	843.0	842.92	1/2*[M+2H]$^{2+}$	ESI+

Supplementary Table. 1. **Mass spectral characterization used for confirmation of successful stapled peptide synthesis.** Each peptide (1-19) used in this inquiry is tabulated by its UA database identifier, synonymous sequence, and exact mass. Theoretical spectral ions, calculated by their assignment formulae, compare with excellent accuracy to observed values. Most peptides were sampled by positive-polarity ESI, although MALDI was used for peptides 1928 and 2011. A single LC/MS run (n=1) was performed for each analyte.

	FAT_{919}- 1907 (6PW8)
**Data Collection**	
Space Group	P 2_1 2_1 2_1
Cell Dimensions	
a, b, c (Å)	47.49, 53.09, 53.42
α, β, γ (°)	90, 90, 90
Resolution (Å)	47.49 - 1.62 (1.65 - 1.62)
R_{merge}	0.159 (17.5)
$I/\sigma I$	8.1 (0.1)
Completeness (%)	90.3 (38.9)
Redundancy	7 (1.7)
**Refinement**	
Resolution (Å)	37.66 - 1.95
No. Reflections	10289
R_{work} / R_{free}	0.2172 / 0.2495
No. Atoms	
Protein	1070
Water	92
Other	27
B-factors	
Protein	37.47
Water	40.92
Other	51.31
R.m.s Deviations	
Bond Lengths (Å)	0.010
Bond Angles (°)	1.123

Number of xtals used in FAT_{919}- 1907 data-set: 1
* Values in parentheses are for highest-resolution shell.

Supplementary Table 2. Data collection and refinement statistics (molecular replacement) of FAT-1907 X-ray co-crystal structure (PDB 6PW8) [https://doi.org/10.2210/pdb6PW8/pdb].

	FAT				1907			Distance	aaProperty	
Res Name	Res ID	Atom	Chain	Res Name	Res ID	Atom	Chain	(Å)	FAT	1907
LEU	965	CD1	A	ASN	1	O	B	3.94	phob	phil
GLU	984	O	A	ASN	1	ND2	B	3.65	phil	phil
GLN	987	CB	A	ASN	1	ND2	B	3.90	phil	phil
LYS	988	CA	A	ASN	1	OD1	B	3.25	phil	phil
ASN	991	ND2	A	ASN	1	O	B	2.87	phil	phil
ARG	962	NH2	A	LEU	2	CD1	B	3.78	phil	phob
LEU	965	CB	A	LEU	2	CD1	B	4.00	phob	phob
ASP	969	OD1	A	LEU	2	CD2	B	3.68	phil	phob
ASN	991	ND2	A	GLU	4	CG	B	3.70	phil	phil
LEU	961	CG	A	LEU	5	CD2	B	3.63	phob	phob
ARG	962	CA	A	LEU	5	CD2	B	3.39	phil	phob
LEU	965	CD1	A	LEU	5	CD2	B	3.59	phob	phob
ASN	991	OD1	A	LEU	5	CB	B	3.32	phil	phob
LEU	994	CD2	A	LEU	5	CD1	B	3.31	phob	phob
ARG	962	NH1	A	ASP	6	OD2	B	2.98	phil	phil
ASN	991	O	A	LEU	8	CD1	B	3.34	phil	phob
GLY	995	N	A	LEU	8	CD1	B	3.67	phob	phob
ILE	998	CD1	A	LEU	8	O	B	3.73	phob	phob
GLY	958	CA	A	LEU	9	CD2	B	3.84	phob	phob
LEU	959	CD2	A	LEU	9	CD1	B	3.63	phob	phob
LEU	994	CD2	A	LEU	9	CD2	B	3.96	phob	phob
LYS	1002	NZ	A	GLU	11	OE2	B	2.80	phil	phil
VAL	951	CG1	A	LEU	12	O	B	3.71	phob	phob
VAL	954	CG1	A	LEU	12	CD2	B	3.72	phob	phob
LYS	955	CA	A	LEU	12	CD1	B	3.91	phil	phob
LYS	955	NZ	A	ASN	13	OD1	B	3.24	phil	phil

Supplementary Table 3. FAT-1907 (6PW8) [https://doi.org/10.2210/pdb6PW8/pdb] interactions ≤ 4 Å at the protein-peptide interface.

FAT				LD2				Distance (Å)	aaProperty	
Res Name	Res ID	Atom	Chain	Res Name	Res ID	Atom	Chain		FAT	LD2
ARG	962	CA	C	LEU	5	CD2	F	3.91	phil	phob
LEU	965	CD1	C	LEU	5	CD2	F	3.80	phob	phob
ASN	991	OD1	C	LEU	5	CD1	F	3.35	phil	phob
LEU	994	CD2	C	LEU	5	CD1	F	3.48	phob	phob
ARG	962	NH2	C	ASP	6	OD1	F	3.11	phil	phil
ILE	998	CD1	C	LEU	8	CB	F	3.76	phob	phob
LYS	955	NZ	C	LEU	9	O	F	3.23	phil	phob
GLY	958	O	C	LEU	9	CD2	F	3.44	phob	phob
ARG	962	CD	C	LEU	9	CD1	F	3.95	phil	phob
ILE	998	CG2	C	GLU	11	OE1	F	3.63	phob	phil
LYS	1002	NZ	C	GLU	11	OE2	F	2.53	phil	phil
VAL	951	O	C	LEU	12	CD2	F	3.59	phob	phob
VAL	954	CG1	C	LEU	12	CD1	F	3.73	phob	phob
LYS	955	CA	C	LEU	12	CD1	F	3.17	phil	phob
MET	1001	CE	C	LEU	12	CD2	F	3.41	phob	phob
LYS	955	NZ	C	ASN	13	OD1	F	3.15	phil	phil

Supplementary Table 4. FAT-LD2 (1OW8) [https://doi.org/10.2210/pdb1OW8/pdb] interactions ≤ 4 Å at the protein-peptide interface.

a

Target	2012	Defactinib	Target	2012	Defactinib
Gene Symbol	% Ctrl @ 1 μM	% Ctrl @ 1 μM	Gene Symbol	% Ctrl @ 1 μM	% Ctrl @ 1 μM
ABL1 (E255K)-phosphorylated	70	82	KIT(V559D,T670I)	93	37
ABL (T315I)-phosphorylated	100	4.9	LKB1	85	90
ABL1-nonphosphorylated	56	37	MAP3K4	90	69
ABL1-phosphorylated	80	51	MAPKAPK2	100	100
ACVR1B	81	83	MARK3	91	50
ADCK3	100	93	MEK1	94	83
AKT1	100	100	MEK2	79	64
AKT2	93	95	MET	100	15
ALK	100	32	MKNK1	100	100
AURKA	100	3.9	MKNK2	100	100
AURKB	100	13	MLK1	100	6.7
AXL	74	3.6	p38-alpha	98	100
BMPR2	100	25	p38-beta	98	89
BRAF	100	100	PAK1	100	65
BRAF (V600E)	100	100	PAK2	100	45
BTK	100	100	PAK4	100	7.4
CDK11	87	97	PCTK1	100	48
CDK2	88	63	PDGFRA	100	82
CDK3	89	62	PDGFRB	94	18
CDK7	69	22	PDPK1	99	50
CDK9	100	90	PIK3C2B	100	100
CHEK1	98	99	PIK3CA	100	100
CSF1R	95	72	PIK3CG	99	85
CSNK1D	97	93	PIM1	100	73
CSNK1G2	99	39	PIM2	100	100
DCAMKL1	86	47	PIM3	100	40
DYRK1B	91	14	PKAC-alpha	91	72
EGFR	93	92	PLK1	89	17
EGFR(L858R)	99	81	PLK3	94	28
EPHA2	98	86	PLK4	97	39
ERBB2	55	65	PRKCE	53	42
ERBB4	83	100	RAF1	81	86
ERK1	100	88	RET	99	12
FAK	96	0.1	RIOK2	91	50
FGFR2	90	43	ROCK2	100	43
FGFR3	95	45	RSK2(Kin.Dom.1-N-terminal)	100	9.9
FLT3	93	1.1	SNARK	100	13
GSK3B	100	75	SRC	99	36
IGF1R	100	71	SRPK3	79	47
IKK-alpha	100	100	TGFBR1	93	91
IKK-beta	100	100	TIE2	98	41
INSR	100	61	TRKA	100	1
JAK2(JH1domain-catalytic)	87	0.1	TSSK1B	98	82
JAK3(JH1domain-catalytic)	83	0	TYK2(JH1domain-catalytic)	77	1.7
JNK1	97	61	ULK2	100	28
JNK2	100	52	VEGFR2	100	37
JNK3	100	90	YANK3	91	89
KIT	96	47	ZAP70	96	95
KIT(D816V)	99	33			

%Ctrl Legend	0≤x<0.1	0.1≤x<1	1≤x<10	10≤x<35	35≤x<50	x≥50

b

Compound Name	Selectivity Score Type	Number of Hits	Number of Non-Mutant Kinases	Screening Concentration (μM)	Selectivity Score
2012	S(35)	0	90	1	0
2012	S(10)	0	90	1	0
2012	S(1)	0	90	1	0
Defactinib	S(35)	23	90	1	0.256
Defactinib	S(10)	11	90	1	0.122
Defactinib	S(1)	3	90	1	0.033

Supplementary Table 5. Eurofins KINOMEscan Profiling. (**a**) The 97 kinases tested against 2012 and defactinib at 1 μM and the resulting screen binding interactions reported as "% Ctrl", where lower numbers indicate stronger hits in the matrix. %Ctrl Calculation = [(test compound signal – positive control signal) /

(negative control signal - positive control signal)] x 100. (**b**) Accompanying Selectivity Scores or S-scores which are quantitative measures of compound selectivity. They are calculated by dividing the number of kinases that compounds bind to by the total number of distinct kinases tested, excluding mutant variants. *S(35)*= (number of non-mutant kinases with %Ctrl <35)/(number of non-mutant kinases tested)], *S(10)*= (number of non-mutant kinases with %Ctrl <10)/(number of non-mutant kinases tested)], *S(1)*= (number of non-mutant kinases with %Ctrl <1)/(number of non-mutant kinases tested)].
