# Supplementary material for: Structure-based discovery of hydrocarbon-stapled paxillin peptides that block FAK scaffolding in cancer
Source: Nat Commun. 2025 Feb 28;16:2060. doi: 10.1038/s41467-025-57196-9 (PMC11871066; doi:10.1038/s41467-025-57196-9)
Supplement: Supplementary file 2 — Reporting Summary [file 41467_2025_57196_MOESM2_ESM.pdf]

nature portfolio

Corresponding author(s): Timothy Marlowe

Last updated by author(s): Feb 6, 2025

Reporting Summary

Nature Portfolio wishes to improve the reproducibility of the work that we publish. This form provides structure for consistency and transparency in reporting. For further information on Nature Portfolio policies, see our Editorial Policies and the Editorial Policy Checklist.

Statistics

For all statistical analyses, confirm that the following items are present in the figure legend, table legend, main text, or Methods section.

n/a	Confirmed	
☐	☒	The exact sample size (n) for each experimental group/condition, given as a discrete number and unit of measurement
☐	☒	A statement on whether measurements were taken from distinct samples or whether the same sample was measured repeatedly
☐	☒	The statistical test(s) used AND whether they are one- or two-sided<br>*Only common tests should be described solely by name; describe more complex techniques in the Methods section.*
☐	☒	A description of all covariates tested
☐	☒	A description of any assumptions or corrections, such as tests of normality and adjustment for multiple comparisons
☐	☒	A full description of the statistical parameters including central tendency (e.g. means) or other basic estimates (e.g. regression coefficient) AND variation (e.g. standard deviation) or associated estimates of uncertainty (e.g. confidence intervals)
☐	☒	For null hypothesis testing, the test statistic (e.g. F, t, r) with confidence intervals, effect sizes, degrees of freedom and P value noted<br>*Give P values as exact values whenever suitable.*
☒	☐	For Bayesian analysis, information on the choice of priors and Markov chain Monte Carlo settings
☒	☐	For hierarchical and complex designs, identification of the appropriate level for tests and full reporting of outcomes
☒	☐	Estimates of effect sizes (e.g. Cohen's d, Pearson's r), indicating how they were calculated

Our web collection on statistics for biologists contains articles on many of the points above.

Software and code

Policy information about availability of computer code

Data collection	BMG ClarioStar MARS software (version 4.20), BioTek Gen 5 (version 3.16), Bruker TopSpin (3.6.3), Unicorn version 5.0, OpenLab CDS ChemStation version 3.0
Data analysis	PyMol (version 1.8), GraphPad Prism (version 10.3.1), Qdat (version 4.3.2 build 8), BioTek Gen 5 (version 3.16), Mestrelab Research MestReNova (v14.2.0), Bruker TopSpin (3.6.3), Spectra Manager (2.0), CCP (version 7.0) XDS (2019 version), OpenLab CDS ChemStation version 3.0, Unicorn version 5.0, ImageJ (version 1.52a), PKSolver (version 2.0)

For manuscripts utilizing custom algorithms or software that are central to the research but not yet described in published literature, software must be made available to editors and reviewers. We strongly encourage code deposition in a community repository (e.g. GitHub). See the Nature Portfolio guidelines for submitting code & software for further information.

Data

Policy information about availability of data

All manuscripts must include a data availability statement. This statement should provide the following information, where applicable:
- Accession codes, unique identifiers, or web links for publicly available datasets
- A description of any restrictions on data availability
- For clinical datasets or third party data, please ensure that the statement adheres to our policy

All data generated or analyzed during this study are included in this published article, source data, and supplementary information files. X-ray crystallographic information generated from this report are available in the Protein Data Bank repository (PDB ID 6PW8).

Human research participants

Policy information about studies involving human research participants and Sex and Gender in Research.

Reporting on sex and gender	N/A
Population characteristics	N/A
Recruitment	N/A
Ethics oversight	N/A

Note that full information on the approval of the study protocol must also be provided in the manuscript.

Field-specific reporting

Please select the one below that is the best fit for your research. If you are not sure, read the appropriate sections before making your selection.

☒ Life sciences ☐ Behavioural & social sciences ☐ Ecological, evolutionary & environmental sciences

For a reference copy of the document with all sections, see nature.com/documents/nr-reporting-summary-flat.pdf

Life sciences study design

All studies must disclose on these points even when the disclosure is negative.

Sample size	Sample sizes for each experiment includes at least 3 biological replicates (n=3) with independently generated samples assayed on separate days. Each biological replicate includes triplicate technical replicates (or more). Based on power calculation of 0.8, standard deviation of group, alpha of 0.05, and treatment effect size, the sample size is sufficient. The exceptions are the LC-MS data (n=1), trypsin protease data (n=1), CD data (n=1), 2020 invasion data (n=2), 2010 SPR data (n=2), and microsome stability (n=2). No statistical tests were made when n < 3.
Data exclusions	Data obtained from experiments with noted experimental/user error (e.g. pipetting error) or non-adherence to assay protocols were not included. All other data is included.
Replication	Experimental optimizations were carefully documented and implemented into final experimental protocols. For publication, these protocols must be strictly followed when completing the experiment and passing of positive/negative controls/QC parameters (Std Dev, assay window) is required. When following finalized protocols without experimental/user error, all attempts at replication were successful.
Randomization	For the in vivo mouse melanoma model, mice were randomized into treatment and control groups (n=8 per group).
Blinding	Investigators were single-blinded to treatment group identification until end of mouse study

Reporting for specific materials, systems and methods

We require information from authors about some types of materials, experimental systems and methods used in many studies. Here, indicate whether each material, system or method listed is relevant to your study. If you are not sure if a list item applies to your research, read the appropriate section before selecting a response.

Materials & experimental systems

n/a	Involved in the study
☐	☒ Antibodies
☐	☒ Eukaryotic cell lines
☒	☐ Palaeontology and archaeology
☐	☒ Animals and other organisms
☒	☐ Clinical data
☒	☐ Dual use research of concern

Methods

n/a	Involved in the study
☒	☐ ChIP-seq
☒	☐ Flow cytometry
☒	☐ MRI-based neuroimaging

Antibodies

Antibodies used

Immunofluorescence staining assay: anti-FAK (clone 4.47) Alexa Fluor 555 conjugate antibody (EMD Millipore; Cat. #116-234; 1:500 dilution); anti-Paxillin (clone B2) Alexa Fluor 647 conjugate antibody (Santa Cruz Biotechnology; Cat # sc-365379 AF647; 1:200); anti-Phalloidin Alexa Fluor 488 conjugate (Cytoskeleton; Cat # PHDG1A; 1:140 dilution)

Validation

Application for anti-FAK antibody cited via Millipore Sigma website as follows: "Detect FAK using this Anti-FAK Antibody, clone 4.47, Alexa Fluor 488 conjugate validated for use in FC, WB & IC." As described on the CoA, 2ug/ml of this antibody showed positive immunostaining for FAK in mouse embryonic fibroblast 3T3 cells. Included negative controls: Catalog # 16-240, Alexa Fluor® 488-conjugated Normal Mouse IgG. Species cross reactivity listed as human, mouse, and rat.
Application for anti-paxillin antibody cited via Santa Cruz Biotechonology website as follows: "paxillin Antibody (B-2) is recommended for detection of paxillin isoforms α, β and γ of mouse, rat and human origin by WB, IP, IF and ELISA; also reactive with additional species, including and equine and canine." As described on the CoA this antibody showed positive immunofluorescence staining for paxillin of methanol-fixed HeLa cells showing cytoplasmic localization and direct immunofluorescence staining of formalin-fixed SW480 cells showing focal adhesions, membrane and cytoplasmic localization.
Application for Phalloidin antibody cited via Santa Cruz Biotechonology website as follows: "Fluorescent staining of actin filaments in fixed tissue sections and tissue culture cells preparations."

Eukaryotic cell lines

Policy information about cell lines and Sex and Gender in Research

Cell line source(s)

HEK293T - ATTC, sex female (Cat #CRL-3216)
SK-BR-3 - ATTC, sex female (Cat #HTB-30)
SK-MEL-147 - Memorial Sloan Kettering Cancer Center, sex unknown
WM88- Rockland, sex male (Cat #WM88-01-0001)
ONDA7- JCRB, sex male (Cat #JCBR1575)
LX2-Millopore Sigma, sex male (Cat #SCC064)
NHEM-PomoCell, sex unknown (Cat # C-12413)
HepG2- ATCC, sex male (Cat #HB-8065)
B16F10- ATCC (Cat #CRL-6475)

Authentication

Cell lines (SK-MEL-147, HEK293T, and SK-BR-3) were cultured in lab, pelleted at 1x10^6 cells, and sent on ice packs to the University of Arizona Genetics Core (UAGC) for authentication. Cell line HEK293T, SK-BR-3, and LX-2 were authenticated via STR profile reference matches available to UAGC. STR profile for SK-MEL-147 produced by UAGC was sent to correspondents of Memorial Sloan Kettering Cancer Center Antibody and Bioresource core for confirmed authentication. LX-2 cells were authenticated and purchased from MilliporeSigma (Cat #SCC064). WM88 cells were authenticated and purchased from Rockland (Cat #WM88-01-0001). ONDA7 cells were authenticated and purchased from JCBR (Cat #JCBR1575). All cells were limited to 10 passages and used within 6 months of authentication.

Mycoplasma contamination

All cell lines tested negative for mycoplasma contamination via MycoAlert™ Mycoplasma Detection Kits, Lonza - LT07-418

Commonly misidentified lines
(See ICLAC register)

No commonly misidentified cell lines were used in this study.

Animals and other research organisms

Policy information about studies involving animals; ARRIVE guidelines recommended for reporting animal research, and Sex and Gender in Research

Laboratory animals

C57Bl/6J female mice, 8-10 weeks of age; Balb/cJ mice, 8-10 weeks of age

Wild animals

N/A

Reporting on sex

Due to ease of use and availability at the UA Experimental Mouse Shared Resources, we have elected to evaluate efficacy and PK in female mice only at this proof-of-concept stage of pre-clinical drug development. Future studies will be performed in both male and female mice to fully investigate the potential of sex as a variable in the efficacy of FAK inhibitor lead peptides.

Field-collected samples	N/A
Ethics oversight	All procedures were approved by the University of Arizona Institutional Animal Care and Use Committee (IACUC)

Note that full information on the approval of the study protocol must also be provided in the manuscript.
